# Comprehensive Analysis of the Membrane Phosphoproteome Regulated by Oligogalacturonides in *Arabidopsis thaliana*

**DOI:** 10.3389/fpls.2016.01107

**Published:** 2016-08-02

**Authors:** Benedetta Mattei, Francesco Spinelli, Daniela Pontiggia, Giulia De Lorenzo

**Affiliations:** Dipartimento di Biologia e Biotecnologie Charles Darwin, Istituto Pasteur - Fondazione Cenci Bolognetti, Sapienza University of RomeRome, Italy

**Keywords:** oligogalacturonides, *Arabidopsis thaliana*, elicitors, DAMPs, phosphoproteomics, immunity, LC-MS/MS, 2DE

## Abstract

Early changes in the *Arabidopsis thaliana* membrane phosphoproteome in response to oligogalacturonides (OGs), a class of plant damage-associated molecular patterns (DAMPs), were analyzed by two complementary proteomic approaches. Differentially phosphorylated sites were determined through phosphopeptide enrichment followed by LC-MS/MS using label-free quantification; differentially phosphorylated proteins were identified by 2D-DIGE combined with phospho-specific fluorescent staining (phospho-DIGE). This large-scale phosphoproteome analysis of early OG-signaling enabled us to determine 100 regulated phosphosites using LC-MS/MS and 46 differential spots corresponding to 34 pdhosphoproteins using phospho-DIGE. Functional classification showed that the OG-responsive phosphoproteins include kinases, phosphatases and receptor-like kinases, heat shock proteins (HSPs), reactive oxygen species (ROS) scavenging enzymes, proteins related to cellular trafficking, transport, defense and signaling as well as novel candidates for a role in immunity, for which elicitor-induced phosphorylation changes have not been shown before. A comparison with previously identified elicitor-regulated phosphosites shows only a very limited overlap, uncovering the immune-related regulation of 70 phosphorylation sites and revealing novel potential players in the regulation of elicitor-dependent immunity.

## Introduction

Plants have developed various mechanisms to defend themselves against biotic stresses. Inducible immune defense responses include phytoalexin accumulation, expression of pathogenesis-related proteins, production of ROS, and, in some cases, programmed cell death (Boller and Felix, 2009). Plant innate immunity is driven by the perception of danger signals mediated by recognition proteins (Chisholm et al., 2006). Pathogen-associated molecular patterns (PAMPs) are conserved molecules secreted or present on the surface of most strains of a given microbial taxonomic group that activate the so-called PAMP-triggered immunity (PTI) against a wide range of pathogens (Barrett and Heil, 2012). Plant immunity also relies on the ability to sense danger by means of endogenous molecular patterns that are present only when the tissue is infected or damaged (damage-associated molecular patterns or DAMPs). In these cases, the discrimination between an intact self and an altered self leads to the activation of the immune system (Benedetti et al., 2015). Recognition of both PAMPs and DAMPs is mediated by the so-called pattern recognition receptors (PRRs; Boller and Felix, 2009).

Oligogalacturonides (OGs) are typical plant DAMPs (Ferrari et al., 2013) produced by the action of fungal *endo*-polygalacturonases (PGs) on homogalacturonan (HGA), the main component of pectin (Bellincampi et al., 2014). The interaction between PGs and the plant polygalacturonase-inhibiting proteins (PGIPs) favors the formation of OGs with degree of polymerization from 10 to 15 that activate the plant innate immunity (Mattei et al., 2005; Casasoli et al., 2009; Kalunke et al., 2015). OGs induce accumulation of phytoalexins, glucanase, and chitinase, ROS production [mediated, in *Arabidopsis thaliana*, by the RESPIRATORY BURST OXIDASE HOMOLOG D (RBOHD)] and callose deposition (Galletti et al., 2008; Ferrari et al., 2013). Exogenous treatment with OGs protects Arabidopsis and grapevine (*Vitis vinifera*) leaves against infection with the necrotrophic fungus *Botrytis cinerea* (Aziz et al., 2004; Ferrari et al., 2007), suggesting that, when PGs are secreted by microbes at the site of infection, this elicitor is likely to be released and contributes to activate defenses responses. Because pectin is one of the most accessible targets for many microbial cell wall–degrading enzymes and among the first structures to be altered during an attempted pathogenic attack, the signaling activity of OGs is an indication that plants have evolved mechanisms to monitor HGA degradation for the early detection of tissue injury (De Lorenzo et al., 2011; Nuhse, 2012; Savatin et al., 2014b). Wall-associated kinase (WAK) receptors are potential candidates to monitor pectin integrity (De Lorenzo et al., 2011; Kohorn and Kohorn, 2012). Indeed, WAK1 has been shown to mediate the perception of OGs (Brutus et al., 2010; Gramegna et al., 2016). OGs may also regulate plant growth and development mainly through their antagonism with auxin (Savatin et al., 2011; Ferrari et al., 2013), demonstrating the potential of this molecule to modulate both developmental and defense-dependent signaling.

Perception of elicitors at the plasma membrane (PM) triggers an intracellular signaling cascade that initiates pathogen defense. Early responses induced by OGs and flg22, a peptide PAMP derived from the bacterial flagellin, largely overlap (Denoux et al., 2008). For example, both activate calcium-dependent protein kinase (CDPK; Gravino et al., 2015) and mitogen-activated protein (MAP) kinase cascades (Rasmussen et al., 2012). The MAP triple kinases indicated as Arabidopsis NUCLEUS- AND PHRAGMOPLAST-LOCALIZED KINASE1-related protein kinases (ANPs), and the MAP single kinases MPK3 and MPK6 play a role in the response to OGs and PAMPs, including the bacterial elicitors flg22 and elf18 (Galletti et al., 2011; Savatin et al., 2014a). Nonetheless, distinctive features have been described between responses induced by PAMPs and OGs. Microarray analyses show considerable differences in the late responses to these two classes of elicitors (Denoux et al., 2008), and lack of ANPs strongly reduces phosphorylation of MPK3 and MPK6 induced by OGs but enhances that induced by elf18 (Galletti et al., 2011; Savatin et al., 2014a). Moreover, while the Arabidopsis leucine-rich repeat co-receptors BAK1/SERK3 and BKK1/SERK4 are required to achieve full response to elf18 and flg22, only a subset of defense responses induced by OGs is affected by loss of these elements, pointing to a complexity in the OG signaling pathways that is unique among the characterized MAMPs and DAMPs (Gravino et al., 2016). Specific phosphorylation events might regulate the activation of distinct signaling branches leading to different downstream responses.

In previous works, several OG-regulated proteins in the Arabidopsis apoplast and nucleus have been identified by 2-D DIGE (Casasoli et al., 2007, 2008), many of them present in multiple isoforms likely due to post-translational modifications (PTMs). Here we investigated early phosphorylation events regulated by OGs in a large-scale phosphoproteomic study including membrane proteins, to facilitate the detection of low abundance proteins with a signaling role. Analysis of membrane proteins by 2-D gel electrophoresis is limited by solubility constraints, that have a minor impact on shotgun proteomics (Kleffmann et al., 2007). On the other hand, greater proteome coverage can be reached by using gel-based and gel-free methods as complementary strategies (Zhao et al., 2008; Robbins et al., 2013). For a comprehensive picture of the phosphoproteome we therefore used both LC-MS/MS and the combination of 2-D DIGE with ProQ Diamond staining, which is known to selectively stain phosphoproteins (hereon indicated as Phospho-DIGE; Bond et al., 2011; Liu et al., 2015). In the latter, phosphorylated isoforms are particularly well resolved due to the property of the acidic phosphate group(s) to lower the pI of the proteins, thereby facilitating the detection of low-abundance phospho-isoforms.

## Materials and methods

### Growth conditions and plant treatments

*A. thaliana* ecotype Columbia-0 (Col-0) was used for this study. Plants were grown on soil (Einheitserde, Germany) in a climatic chamber at 22°C and 70% relative humidity. Seedlings were grown in a growth chamber at 21°C. Sterilized seeds (20 per well) were germinated and grown in 1 mL of liquid growth medium [Murashige and Skoog (MS) medium, pH 5.7, 0.5% sucrose] in 12-well plates.

For proteomic analyses, seedlings were grown in 500-ml flasks containing 100 mL of growth medium. Flasks containing seeds (about 100 seeds per flask) were grown in a growth chamber at 21°C. After 2 weeks, the culture medium was replaced with fresh medium and seedlings were grown for an additional day. About 10 g (fresh weight) of plant material were obtained from each flask. Plants and seedlings were grown under a 16-h light/8-h dark cycle (~120 μmol m^−2^ s^−1^).

OGs with degree of polymerization of 9–16 were prepared as previously described (Pontiggia et al., 2015). The OG stock solution (10 mg/mL) was filter-sterilized before addition to the medium to a final concentration of 50 μg/ml. For proteomic studies, water- (control), and OG-treated seedlings were harvested 10 min after treatment for protein extraction.

### Preparation of total protein extract and total microsomal fraction

To obtain total protein extracts for Phospho-DIGE analysis, 10 g seedlings were homogenized using mortar and pestle in liquid nitrogen in homogenization buffer (1 M NaCl, 1 mM Na_3_VO_4_, 1 mM Na_2_MoO_4_, 25 mM NaF, protease inhibitor cocktail, Sigma). Total microsomal fractions (TMF) for both Phospho-DIGE and LC-MS/MS analyses were prepared as previous reported (Alexandersson et al., 2004; Tang et al., 2008). Briefly, 10 g seedlings were grinded using a blender with three impulses of 10 s in lysis buffer containing 1 mM Na_3_VO_4_, 1 mM Na_2_MoO_4_, 25 mM NaF, protease inhibitor cocktail (Sigma). The mixture was filtered through Miracloth and centrifuged at 10,000 × g for 10 min. Supernatant was then centrifuged at 100,000 × g for 1 h and the pellet (TMF) was recovered.

### H^+^-ATPase immunoblotting

Protein samples (4 μg each) were boiled for 3 min in Laemmli buffer. Following gel electrophoresis, proteins were blotted onto nitrocellulose membranes using a Trans-Blot Turbo apparatus (BioRad). Subsequently blot was first incubated with the antibody against the plasma membrane H^+^-ATPase (1:500 dilution in PBS-T, kind gift of Prof M.I. De Michelis, University of Milan) and then with a horseradish peroxidase-coupled secondary antiserum (Santa Cruz, Biotechnology, Santa Cruz, CA; 1:2000 dilution in PBS-T).

### MPK3/MPK6 immunoblotting

Immunodetection of phosphorylated MAPKs MPK3 and MPK6 was performed using a polyclonal phospho-p44/42-ERK MAPK-specific antiserum (Cell Signaling Technology, Danvers, MA). Protein samples (30 μg each) were boiled for 3 min in Laemmli buffer. Following gel electrophoresis, proteins were blotted onto nitrocellulose membranes using a Trans-Blot Turbo apparatus (Biorad). Subsequently blots were first incubated with the phospho-p44/42 MAPK-specific antiserum (1:2000 dilution in TBS-T at 4°C overnight) and then with a horseradish peroxidase-coupled goat anti-rabbit secondary antiserum (Santa Cruz, Biotechnology, Santa Cruz, CA; 1:5000 dilution in PBS-T). Membrane was stripped and re-probed with anti-MAPK3 and anti-MAPK6 (Sigma) and developed as reported above.

### Protein digestion and phosphopeptide enrichment for LC-MS/MS analysis

TMF from control and OG-treated samples of two independent biological replicates were resuspended in 100 μL of freshly prepared 8 M urea, 10 mM Tris-HCl pH 8.0, 5% sodium deoxycolate. Protein concentration was determined using the BCA Protein Assay Kit (Sigma). For each sample, 150 ug of proteins were subjected to reduction with 10 mM dithioreitol for 45 min at 56°C followed by alkylation of cysteines with 50 mM iodoacetamide for 25 min at room temperature in darkness. Proteolytic digestion was carried out overnight with proteomics grade Lys-C (Promega, Lys-C:protein ratio 1:50) and for additional 4 h with trypsin (Promega, trypsin:protein ratio 1:50) at room temperature. The digestion mixture was subsequently acidified with 1% (v/v) formic acid and centrifuged to remove insoluble material. Peptides were desalted using home-made microcolumns using R3 beads (Poros) packed in gel loader tips. Phosphopeptide enrichment was performed using a sequential elution from immobilized metal affinity chromatography (SIMAC) as previously described (Thingholm et al., 2008). The procedure includes an initial phosphopeptide enrichment step by IMAC, in which bound peptides are sequentially eluted with an acidic and a basic solution. Next, the IMAC flow-through fraction and the acidic elution fractions are subjected to a second phosphopeptide enrichment step using TiO_2_ chromatography. The TiO_2_-enriched fraction and the basic elution fractions from IMAC were lyophilized and analyzed by LC-MS/MS. For proteomic analysis, also flow-through fractions from TiO_2_ chromatography were desalted on home-made R3 stage tips and analyzed by LC-MS/MS.

### Proteomic and phosphoproteomic analysis by LC-MS/MS

Peptides were separated on a 15 cm PicoFrit Column (75 μm i.d., New Objective, Woburn, MA) packed with Magic C18AQ (5 μm, 200 Å, Michrom) and analyzed on a LTQ-Orbitrap Discovery (Thermo Fisher Scientific) coupled on-line with a nano-HPLC (Ultimate 3000, Thermo Fisher Scientific). Peptides were eluted using a 0–60% acetonitrile gradient in 0.1% formic acid (180 min, 300 nl/min). MS was acquired at 30,000 FWHM resolution in the FTMS (target value of 5 × 10^5^ ions) and MS/MS was carried out in the linear ion trap, with MS/MS on top 5 ions and multistage activation.

### LC-MS/MS data analysis

MS data were processed using MaxQuant (Cox et al., 2014) version 1.5.2.8 with a false-discovery rate (FDR) < 0.01 at the level of proteins, peptides and modifications, using default settings. Oxidized methionine, acetylation (protein N-term) and phospho(STY) were selected as variable modifications, and carbamidomethyl cysteine as fixed modification. Proteins were identified using a target-decoy approach with a reversed database, using the Andromeda search engine against the Arabidopsis UniProt database (release-2014, 31565 entries) and LFQ quantification for phosphoproteomics and proteomics was performed by MaxQuant.

Statistical analysis was performed with Perseus (version 1.5.0.31). Hits to the reverse database and contaminants were filtered out, intensities were normalized for unequal protein amounts and log_2_-transformed. For proteomic analysis quantification was performed on proteins identified with a minimum of 2 unique peptides and at least three valid values among the replicates. Significance was assessed by Student's *t*-test, using permutation-based FDR to control for multiple hypothesis testing (Data Sheet S2B in Supplementary Material). For the phosphoproteomic study, we first filtered to retain only class I phosphosites (localization probability > 0.75 and score difference > 5). We next considered (1) phosphosites that were exclusive for either the OG-treated samples or the control samples with two valid values in either the control or the OG-treated group; (2) phosphosites that had at least three valid values (control + OG-treated). These sites are listed in Data Sheet S1B in Supplementary Material. For phosphosites that had three valid values only, first, missing values were imputed with values representing a normal distribution around the detection limit of the mass spectrometer (downshift = 1.5; width = 0.3), to allow statistical analysis; then Significance *B*-values for each replicate (OG-treated vs. control) were calculated using the statistical tool in Perseus (Cox and Mann, 2008). Significance *B*-values represent outlier probability score weighted for the intensity. Besides the exclusive ones, phosphosites with a significance B *p* ≤ 0.05 in both replicates were considered as significantly regulated. In addition, a two-sample student's *t*-test was performed, and phosphosites that had a significance *B* ≤ 0.05 in one replicate and passed the *t*-test with *p* < 0.05 were also considered as significantly regulated.

### 2D DIGE, Pro-Q diamond staining, and image acquisition

The Clean-up kit (GE Healthcare) was used to eliminate salts and concentrate proteins in both the total protein extracts and the TMF. The protein-containing pellets were finally dissolved in a small volume (50–100 μL) of IEF buffer containing 8 M urea, 2 M thiourea, and 4% (v/v) CHAPS, centrifuged and the supernatants recovered. Protein content was measured using the BIO-RAD protein assay according to the manufacturer's instructions. Total protein extracts and TMF were labeled and analyzed using the same procedure: control and OG-treated protein samples from 3 independent biological replicates (C1, C2, C3; T1, T2, T3) were labeled with Cy3 or Cy5, according to the manufacturer's instructions for minimal labeling (Minimal labeling kit, GE Healthcare) and randomization (ETTAN DIGE System, GE Healthcare). Briefly, each sample (50 μg) was labeled with 200 pmol CyDye DIGE Fluor minimal dyes (GE Healthcare) and incubated on ice in the dark for 30 min. The internal standard (IS) was labeled with Cy2 and consisted of a pooled sample comprising equal amounts of each sample used for each replicate (C1+C2+C3+T1+T2+T3). Equal volumes of a 2X buffer [8 M urea, 2 M thiourea, 2% ASB-14 (or 4% CHAPS; v/v), 20 mg/mL DTT, and 2% IPG buffer/Pharmalytes 4-7] were added to each labeled protein samples. Samples (C+T+IS) were pooled prior to IEF, which was carried out using non-linear IPG strips (pH 4–7, 13 cm, GE Healthcare) rehydrated overnight at room temperature. IEF was performed using the Ettan IPG-phor apparatus (GE Healthcare) as follows: (1) step to 500 V (0.5 kVh); (2) step to 1000 V (0.8 kVh); (3) step to 8000 V (11.3 kVh); (4) step to 8000 V (3.0 kVh), for a total of 25 kVh at 20°C and a maximum current setting of 50 μA per strip. After IEF, the strips were equilibrated for 15 min in 100 mM Tris pH 6.8, 30% v/v glycerol, 8 M urea, 1% w/v SDS, 0.2 mg/mL bromophenol blue, 5 mg/mL DTT for reduction of disulfide bridges, and alkylated for 15 min in the same equilibration buffer containing 25 mg/mL iodoacetamide. Each strip was then loaded on top of a 12% w/v acrylamide gel for the second dimension. SDS-PAGE was carried out using the Hoefer SE 600 Ruby apparatus (GE Healthcare) at 100 V and 20 mA per gel for 15 min and then 100 V and 40 mA per gel until the bromophenol blue dye front had run off the bottom of the gel. A running buffer of 25 mM Tris pH 8.3, 192 mM glycine, and 0.1% w/v SDS was used.

Each gel was scanned using a Typhoon 9200 imager (GE Healthcare) set at the wavelengths corresponding to each CyDye [532 nm laser and 580 nm band pass (BP) 30 emission filter for Cy3; 633 nm laser and 670 nm BP30 emission filter for Cy5; 488 nm laser and 520 nm BP40 emission filter for Cy2], at high resolution (100 μm). The photo multiplier tube (PMT) voltage was adjusted for each channel between 500 and 700 V to ensure that the image intensity was within a linear range between 40,000 and 60,000 U. For phosphoprotein detection, each gel was then fixed, post-stained with Pro-Q Diamond phosphospecific fluorescent dye (Invitrogen; Bond et al., 2011; Liu et al., 2015) applying the same scanning conditions described above. Pro-Q Diamond and Cy3 have very similar spectra of fluorescence with excitation/emission maxima at 555/580 and 553/569 nm for Pro-Q Diamond and Cy3, respectively. Therefore, phosphoproteins were detected as spots with increased Cy3 fluorescence (Cy3 + Q) in comparison to original Cy3.

### Phospho-DIGE statistical analysis

For data normalization and analysis, gel images acquired before and after Pro-Q Diamond post-staining were compared using the Differential In-gel Analysis (DIA) module of the DeCyder software version 6.5 (GE Healthcare) for co-detection of the three CyDye-labeled forms of each spot and calculation of the ratios between sample and internal standard abundance. Phosphoproteins were detected as spots with increased Cy3-like fluorescence due to ProQ-staining in comparison with original Cy3 emission (Stasyk et al., 2005). Statistical analysis of protein abundance changes between control and treated samples from the three independent biological replicates was performed in the Biological Variation Analysis (BVA) module for quantitative comparisons of protein abundance/phosphorylation across multiple gels. The inter-gel variability was corrected by normalization of the Cy2 internal standard spot map present in each gel. Protein spots whose intensities were significantly different among the control and treated samples were determined by paired one-way ANOVA. Proteins were considered differentially expressed when the FDR-corrected *p*-values of the ANOVA analysis were < 0.05.

### Protein spot identification by MALDI-TOF mass spectrometry

For mass spectrometry analysis of proteins identified as differentially phosphorylated by Phospho-DIGE experiments, a preparative gel was run under the same IEF and SDS-PAGE conditions used for the DIGE gels, loading 250 μg each of control and OG treated samples mixed together. Proteins were subsequently visualized using Coomassie Brilliant Blue (CBB) R-250 stain (Sigma) according to the manufacturer's instructions. The preparative gel image was matched to the master gel image (the gel with the highest spot count in the DIGE analytical gel match-set) using DeCyder software. Matching was further improved by land marking and manually confirming potential spots of interest. By comparing the CBB-stained spot pattern with the corresponding Cy5 protein pattern, spots of interest showing differential fluorescent levels on the 2-D DIGE gels were picked manually from the preparative gel and subjected to trypsin in-gel digestion as previously described (Casasoli et al., 2007).

Protein identification was carried out using a Voyager-DE STR instrument (Applied Biosystems, Framingham, MA). Peptides were desalted using ZipTip C18 microcolumns (Millipore, Bedford, MA, USA) and spotted onto a MALDI target plate using CHCA as the matrix (10 mg/ml α-cyano-4-hydroxy-cinnamic acid in 0.1% TFA, 50% ACN). The mass spectrometer was operated in the positive ion, delayed extraction (200 ns delay time) reflector mode with an accelerating voltage of 20 kV. Each MALDI-TOF spectrum was generated by accumulating data corresponding to 200–500 laser shots. Internal mass calibration was performed by using theoretical masses of the trypsin autodigestion peaks. The mass list was then analyzed using the PeakErazor software (http://www.welcome.to/GPMAW) to eliminate contaminant peaks (keratin, trypsin added for digestion and peaks present in all mass spectra). Proteins were identified by searching the National Center for Biotechnology Information (NCBI) database using the MASCOT (www.matrixscience.com) or Aldente (xpasy.org/tools/aldente) search engines with the following criteria: cleavage by trypsin (one missed cleavage allowed), mass tolerance 30 ppm; carbamidomethyl cysteine as fixed modification, methionine oxidation, and phospho(STY) as variable modifications. Only matches with a Mascot score higher than 60 (*p* < 0.05; or Aldente score higher than 13.86, *p* < 0.05), sequence coverage higher than 10% and more than 6 peptide matches were considered significant. A number of photosynthetic proteins were identified as potentially regulated phosphoproteins. Because they corresponded to highly abundant protein spots amenable to mis-quantification, they were excluded from our dataset.

### Bioinformatic analysis of phosphoproteins

The PhosPhAt (http://phosphat.mpimp-golm.mpg.de/; Durek et al., 2010) and P3DB (http://www.p3db.org/index.php; Yao et al., 2014) databases were searched to determine if phosphorylation sites had been previously reported for the identified proteins. For the analysis of significantly over-represented GO terms, differentially phosphorylated proteins were analyzed using AgriGO (http://bioinfo.cau.edu.cn/agriGO/analysis.php) for Singular Enrichment Analysis (SEA) using *A. thaliana* TAIR10 as selected species and Arabidopsis genome locus (TAIR10) as selected reference, Fisher as a statistical test method, Yekutieli (FDR under dependency) as z multi-test adjustment method, 0.05 as significance level, 5 as minimum number of mapping entries and complete GO as Gene Ontology type. In order to avoid redundancy at the protein level, in the case of protein groups we considered only the first isoform.

To investigate possible interactions between the OG-regulated phosphoproteins, the STRING (Search Tool for the Analysis of Interacting Genes/Proteins) algorithm was used for the creation of protein interaction networks based on published functional or informatics-predicted interactions (Szklarczyk et al., 2015). The evidence annotation in STRING was filtered out of interactions from text-mining and neighborhood, and only interactions supported by experimental evidence, co-expression and existing database information, with high-confidence score >0.7 were considered (Table S6). The SUBA3 database (http://suba3.plantenergy.uwa.edu.au/; Heazlewood et al., 2005) was used to assign the subcellular localization of differentially phosphorylated proteins.

## Results

### OG treatment induces early changes in the phosphoproteome of arabidopsis seedlings

**Figure 1:**
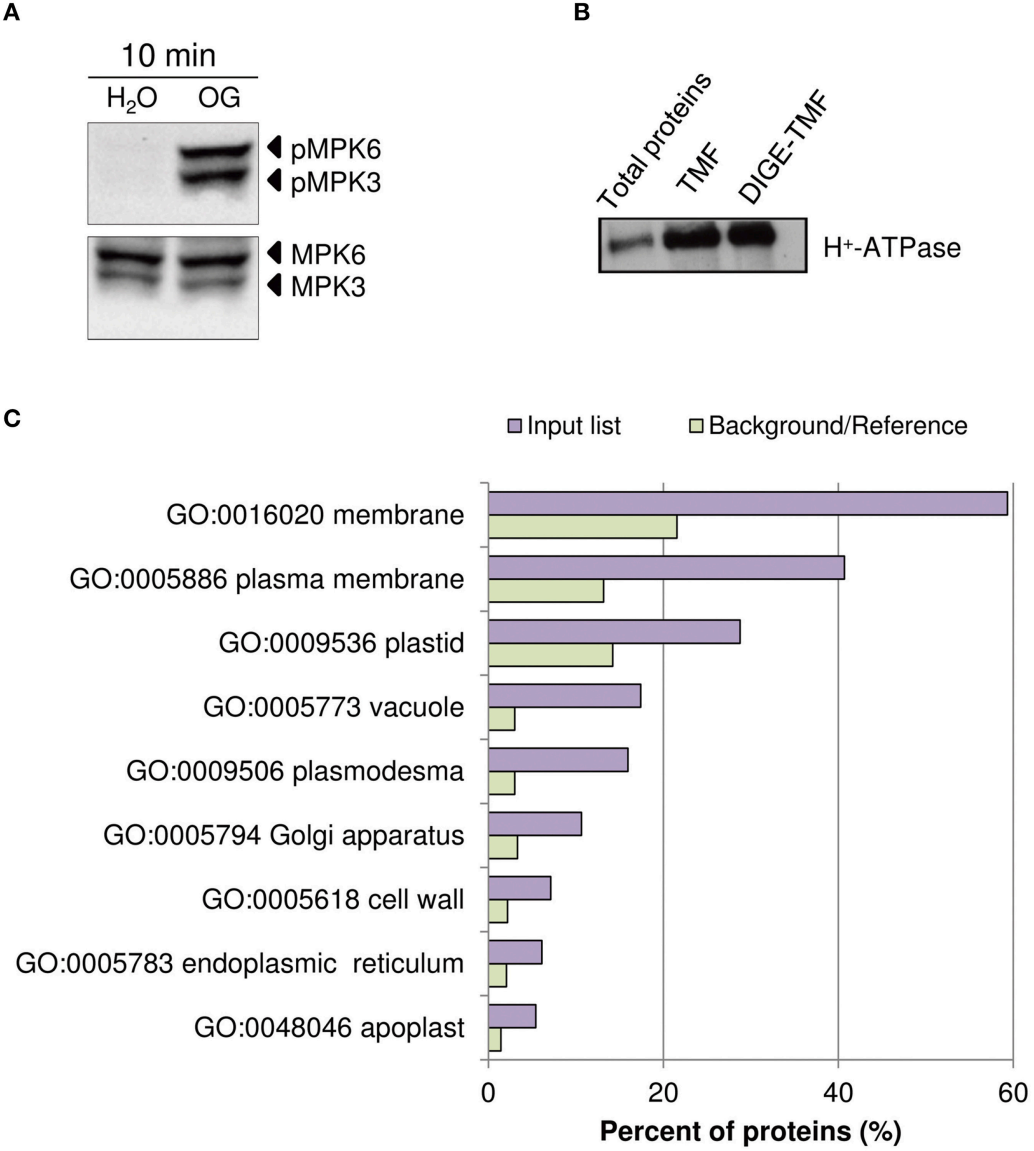
**Assessment of OG treatment efficacy and plasma membrane protein enrichment. (A)** Detection of the phosphorylation of *Arabidopsis thaliana* MAPKs MPK6 and MPK3 upon treatment of seedlings for 10 min with OGs by immunoblot analysis using an anti-p44/42-ERK antibody (top panel). Thirty micrograms of each protein sample were loaded. Levels of MPK3 and MPK6 total proteins were determined using specific antibodies (bottom panel). The identity of individual MAP kinases, as determined by size, is indicated by arrows. **(B)** Western blot analysis of total protein extracts, TMF proteins directly solubilized in Laemmli buffer (TMF) or immediately before DIGE analysis (DIGE-TMF), probed with antibody against the plasma membrane H^+^-ATPase. Four micrograms of each protein sample were loaded. **(C)** The analysis of enriched GO terms for the cellular component category within the TMF proteins identified (listed in Data Sheet S2A in Supplementary Material) using the AgriGO database. Barplot shows selected GO terms significantly enriched (FDR < 0.05, Yekutieli adjusted). The background/reference represents the proportion of all annotated genes of each GO term within the total genes in the TAIR10 database.

In Arabidopsis, treatment with OGs induces the activation, by phosphorylation, of MPK3 and MPK6 within few minutes, a temporal kinetics similar to that observed upon treatment with PAMPs (Galletti et al., 2011). Two-week-old liquid-grown Arabidopsis ecotype Columbia 0 (Col-0) seedlings were treated with OGs (50 μg/ml) or water as a control. The 10-min time point was chosen for our analyses, because close to that used in previous studies on elicitor-induced phosphorylation (Benschop et al., 2007; Nuhse et al., 2007; Rayapuram et al., 2014). Western blot analysis using α-phospho-p44/42-ERK antibody showed the OG-induced phosphorylation of MPK3 and MPK6 (Figure 1A). TMF was obtained to facilitate the identification of less abundant membrane proteins. Enrichment of total membranes was determined by western blot using antibodies specific for the PM H^+^-ATPase (Figure 1B). In addition, total protein extracts were obtained, for Phospho-DIGE analysis only.

### Phosphoproteomic analysis of TMF by LC-MS/MS

TMF preparations from seedlings treated with OGs or water as a control were subjected to the SIMAC phosphopeptide enrichment method. The enriched fractions were analyzed by nano-LC-MS/MS, leading to the identification of a total of 2147 unique phosphosites [Data Sheet S1C in Supplementary Material]. We could quantify 1026 phosphosites (Data Sheet S1B in Supplementary Material). A total of 99 phosphosites significantly changed phosphorylation level upon OG treatment; among them, 29 and 36 phosphosites were unique to control and OG-treated seedlings, respectively (Table 1).

**Table 1:** **List of LC-MS/MS identified phosphosites regulated by OGs^a^**.

**Protein name^b^**	**TAIR ID^c^**	**Loc^d^**	**Uniprot ID^e^**	**Pos^f^**	**Phosphopeptide (best evidence)^g^**	**Average log2 ratio Tr/C^h^**	**Log2 ratio Tr1/C1^i^**	**Log2 ratio Tr2/C2^i^**	**μarray OG 1 h/3 h^j^**
**TRANSPORT**									
Aquaporin PIP2-1; Aquaporin PIP2-2; Aquaporin PIP2-3	At3g53420; At2g37170; At2g37180	PM^§^	P43286; P43287; P30302	280; 278 278	SI G**S**(0.996)FRS(0.004)AANV	Tr	Tr	Tr	ns
ABC transporter B family member 20 (ABC-B20)	At3g55320	PM	Q9M3B9	712	GS(0.001)GVFRPQEICFD**T**(0.978)EES(0.054)PKAHS(0.216)PAS(0.751)EK	Tr	Tr	Tr	ns
Translocase of chloroplast 159 (TOC159)	At4g02510	PL^§^	O81283	686	IDGQIVT(0.003)D**S**(0.996)DEDVDT(0.001)EDEGEEK	Tr	Tr	Tr	ns
ABC transporter C family member 14 (ABCC14)	At3g62700	VO	Q9LZJ5	903	S(0.348)IS(0.077)IES(0.575)PRQPK**S**(1)PK	Tr	Tr	Tr	−1.5/ns
ABC transporter C family member 14 (ABCC14)	At3g62700	VO	Q9LZJ5	925	T(0.333)T(0.333)S(0.333)ME**S**(0.999)PRISEINDESIK	Tr	Tr	Tr	−1.5/ns
ABC transporter G family member 36 (PEN3) 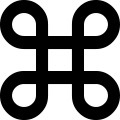	At1g59870	PM^§^	Q9XIE2	45	T(0.011)Q**S**(0.989)VNDDEEAIK	5.70	4.77	6.62	2.8/ns
ABC transporter C family member 14 (ABCC14)	At3g62700	VO	Q9LZJ5	897	S(0.009)IS(0.991)IE**S**(1)PRQPKS(1)PK	4.63	3.24	6.02	−1.5/ns
ABC transporter C family member 14 (ABCC14)	At3g62700	VO	Q9LZJ5	894	S(0.009)I**S**(0.991)IES(1)PRQPKS(1)PK	4.55	3.24	5.87	−1.5/ns
ATPase 1 (AHA1), plasma membrane 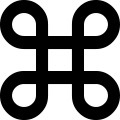	At2g18960	PM^§^	P20649	544	D**S**(1)NIASIPVEEIIEK	4.17	3.83	4.50	ns
Transport protein particle (TRAPP) component	At3g05000	CY	Q9CAW4	121	VSIDPSSENETQDPS**T**(0.999)PGES(0.001)K	3.98	4.17	3.79	ns
ATPase 2 (AHA2), plasma membrane; ATPase 1 (AHA1), plasma membrane 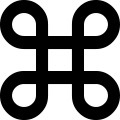	At4g30190; At2g18960	PM^§^	P19456; P20649	899; 899	G**S**(1)YREISEIAEQAK	3.42	3.08	3.77	ns/2.3
Copper transporter 5 (CuTR5)	At5g20650	GO^§^	Q93VM8	68	S(0.018)S(0.018)**S**(0.962)GVS(0.001)APIIPK	C	C	C	ns
UDP-galactose transporter 1	At1g77610	GO	Q9C521	310	HMIS(0.001)QQT(0.006)PG**T**(0.821)PRT(0.821)PRT(0.35)PR	C	C	C	−1.6/ns
UDP-galactose transporter 1	At1g77610	GO	Q9C521	313	HMIS(0.001)QQT(0.006)PG**T**(0.821)PR**T**(0.821)PRT(0.35)PR	C	C	C	−1.6/ns
NSP (Nuclear shuttle protein)-interacting GTPase	At4g13350	CY	Q8W4K6	407	S(0.214)M**S**(0.779)APS(0.007)IQPIQGVPSGGIQSSEVKPSGR	C	C	C	na
NSP (Nuclear shuttle protein)-interacting GTPase	At4g13350	CY	Q8W4K6	219	T(0.045)S(0.045)EEGS(0.185)QS(0.725)PEQVKDIGS(0.199)A**S**(0.801)PPVARPVR	C	C	C	na
**SIGNAL TRANSDUCTION**									
Transcription regulator NOT2/NOT3/NOT5 family protein	At5g18230	NU	F4JWJ6	431	NIMGVESNVQPIT(0.043)**S**(0.891)PIS(0.066)K	Tr	Tr	Tr	ns
Transducin family protein (WD-40 RFP)	At5g05570	MI^§^	F4K0R0	464	AR**T**(0.928)PRT(0.661)PS(0.303)GES(0.107)AQWPIT(0.002)GGVPSHVDDYK	Tr	Tr	Tr	ns
Phospholipase like protein (PEARLI 4f) family	At4g38550	NU	Q9C5F6	232	EQFEDIYEQDGDV**T**(1)PR	Tr	Tr	Tr	6.7/ns
Phototropin-2 (PHOT2)	At5g58140	PM^§^	F4KDJ3	504	GEIQYFIGVQIDGSDHVEPIQNRI**S**(1)ER	Tr	Tr	Tr	ns
Mitogen-activated protein kinase 6 (MPK6)	At2g43790	CY^§^	Q39026	221	VTSESDFM**T**(1)EY(1)VVTR	Tr	Tr	Tr	ns
Mitogen-activated protein kinase 6 (MPK6)	At2g43790	CY^§^	Q39026	223	VTSESDFMT(1)E**Y**(1)VVTR	Tr	Tr	Tr	ns
Calmodulin-binding protein (EDA39)	At4g33050	CY	O82645; F4JVX1	462; 500	VS(0.001)PANS(0.003)Y(0.003)GPIP**S**(0.996)PRPS(0.998)PK	Tr	Tr	Tr	24.1/2.9
Calmodulin-binding protein (EDA39)	At4g33050	CY	O82645; F4JVX1	466; 504	VS(0.001)PANS(0.003)Y(0.003)GPIPS(0.996)PRP**S**(0.998)PK	Tr	Tr	Tr	24.1/2.9
Phospholipase D gamma 1(PLDγ1)	At4g11850	VO	Q9T053	680	EVPVGTVS(0.001)VY(0.014)N**S**(0.984)PR	3.83	4.47	3.20	ns
Protein kinase family protein	At5g03320	PM	Q9LZF8	17	DEQR**S**(1)PKPVS(0.806)PT(0.082)S(0.027)NFS(0.085)DVNK	2.79	3.15	2.43	2.5/ns
Putative tyrosine phosphatase (PTEN2a)	At3g19420	NU	Q9LT75	509	ETENPDKDDVF**S**(1)DNEGDSTGPTK	2.62	2.42	2.81	1.5/ns
Cyclin-dependent protein kinase-like protein	At5g44290	NU	Q9FKV9	75	HQEIAEIGD**T**(1)DEDEDDDHHPPEEIK	−2.67	−2.31	−3.03	2.9/ns
Auxin response factor ARF1	At1g59750	NU	F4ID31	399	PRPPGIPS(0.084)PAT(0.06)GPS(0.163)DGVWKS(0.159)PADT(0.664)P**S**(0.825)S(0.825)VPIFS(0.221)PPAK	−2.74	−2.98	−2.50	ns
Auxin response factor ARF1	At1g59750	NU	F4ID31	400	PRPPGIPS(0.084)PAT(0.06)GPS(0.163)DGVWKS(0.159)PADT(0.664)PS(0.825)**S**(0.825)VPIFS(0.221)PPAK	−2.74	−2.98	−2.50	ns
Serine/threonine protein kinase	At1g48210	PM	F4HWU0; Q9LUT0	353; 354	AIQPIINPPR**S(**0.986)APQT(0.986)PHRNPY(0.027)	C	C	C	ns
Serine/threonine protein kinase	At1g48210	PM	F4HWU0; Q9LUT0	357; 358	AIQPIINPPRS(0.986)APQ**T**(0.986)PHRNPY(0.027)	C	C	C	ns
MAP3K epsilon protein kinase (MKKK7)	At3g13530	PM^§^	Q9LJD8	788	VR**S**(1)GQIDPNNPIFGQNETSSISMIDQPDVIK	C	C	C	ns
Probable LRR receptor-like serine/threonine-protein kinase	At5g37450	PM	C0LGU1	268	**S**(0.886)IVIY(0.059)Y(0.059)IDIS(0.578)S(0.889)NKIT(0.528)GEIPKNK	C	C	C	ns
Probable LRR receptor-like serine/threonine-protein kinase	At5g37450	PM	C0LGU1	278	S(0.886)IVIY(0.059)Y(0.059)IDIS(0.578)**S**(0.889)NKIT(0.528)GEIPKNK	C	C	C	ns
GSK3/Shaggy-related protein kinase alpha (ASK1)	At5g26751	PM	P43288	229	GEPNIS(0.232)**Y**(0.768)ICSR	C	C	C	1.6/ns
Probable serine/threonine-protein kinase	At1g18390	PM	P0C5E2	643	S(0.003)GPIVAQ**S**(0.761)PDS(0.235)VIVK	C	C	C	6.8/1.5
Phospholipase-like protein (PEARLI 4) 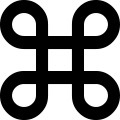	At2g20960	NU	Q9SKR5	314	**S**(0.829)KT(0.169)PEPQPT(0.001)Y(0.001)FEPSSR	C	C	C	4.0/ns
**TRAFFICKING**									
Patellin-2 (PATL2)	At1g22530	PM	Q56ZI2	79	EIIQS(0.064)E**S**(0.936)FK	Tr	Tr	Tr	−2.0/−1.6
Patellin-4 (PATL4)	At1g30690	PM	Q94C59	53	S(0.126)A**S**(0.874)FKEESDFFADIK	Tr	Tr	Tr	ns
ADP-ribosylation factor GTPase-activating protein (ZIGA4)	At1g08680	NU	F4HXP0	236	S(0.001)DIQ**S**(0.999)PNFQQEAEFR	Tr	Tr	Tr	ns
Kinesin-like protein KAC1	At5g10470	CY^§^	Q9LX99	611	TGDAIQS(0.001)QDIF**S**(0.999)PIPK	Tr	Tr	Tr	ns
Kinesin-like protein KAC1	At5g10470	CY^§^	Q9LX99	698	GEGYSAEAVAIPS(0.063)**T**(0.937)PNK	Tr	Tr	Tr	ns
65-kDa microtubule-associated protein 1 (MAP65-1)	At5g55230	NU; CS^§^	F4K3E4; Q9FLP0	615; 586	EEAASSPVSGAADHQVPA**S**(1)P	Tr	Tr	Tr	ns
Myosin-17 (XIK) 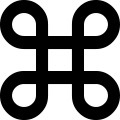	At5g20490	ER^§^	F4K5J2	990	QQAIAI**S**(0.957)PT(0.041)S(0.002)R	3.86	5.22	2.49	ns
Patellin-3 (PATL3)	At1g72160	CY	Q56Z59	108	SMIPQNIG**S**(1)FK	3.30	2.95	3.66	ns
Membrane fusion protein Use1	At3g55600	NU	Q6NKR3	104	IEDEPR**S**(0.999)PT(0.973)S(0.028)PQIR	2.42	2.91	1.94	1.8/ns
Syntaxin of plants 121 (SYP121) 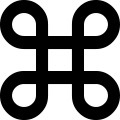	At3g11820	PM^§^; GO^§^	Q9ZSD4	264	AS(0.213)**S**(0.755)FIRGGT(0.031)DQIQTAR	−3.06	−2.37	−3.74	7.5/ns
Syntaxin of plants 122 (SYP122) 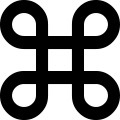	At3g52400	PM^§^	Q9SVC2	18	MIAIFHEAFAHPPEEIN**S**(0.983)PAS(0.017)EK	−4.08	−2.61	−5.54	11.5/ns
**RESPONSE TO STRESS**									
Respiratory burst oxidase homolog protein D	At5g47910	PM^§^	Q9FIJ0	343	II**S**(1)QMIS(1)QK	Tr	Tr	Tr	9.9/ns
Respiratory burst oxidase homolog protein D	At5g47910	PM^§^	Q9FIJ0	347	IIS(1)QMI**S**(1)QK	Tr	Tr	Tr	9.9/ns
Metal tolerance protein C2	At3g12100	VO	F4J8M5	37	**S**(0.99)FQQS(0.212)HGPRT(0.688)PAVT(0.11)EAAKPFIDR	Tr	Tr	Tr	ns
Aluminium induced protein with YGL and LRDR motifs	At5g43830	CY	Q9FG81	18	TVANSPEAIQ**S**(0.997)PHS(0.003)SESAFAIK	5.95	6.83	5.06	ns
Aluminium induced protein with YGL and LRDR motifs	At4g27450	CY	Q93V62	219	VD**S**(1)EGVICGANFK	−2.83	−3.56	−2.10	ns
Protein plant cadmium resistance 8 (CdRes8)	At1g52200	PM	Q9M815	12	GRVT(0.002)T(0.002)PS(0.184)EED**S**(0.812)NNGIPVQQPGT(1)PNQR	C	C	C	6.1/2.3
Putative defensin-like protein 135	At4g09647	EX	Q2V3K1	2	**S**(0.946)KT(0.849)FQPS(0.176)IVMIT(0.036)IFIIIVT(0.996)S(0.997)QR	C	C	C	ns
Putative defensin-like protein 135	At4g09647	EX	Q2V3K1	21	S(0.946)KT(0.849)FQPS(0.176)IVMIT(0.036)IFIIIVT(0.996)**S**(0.997)QR	C	C	C	ns
Putative defensin-like protein 135	At4g09647	EX	Q2V3K1	4	S(0.946)K**T**(0.849)FQPS(0.176)IVMIT(0.036)IFIIIVT(0.996)S(0.997)QR	C	C	C	ns
Putative defensin-like protein 135	At4g09647	EX	Q2V3K1	20	S(0.946)KT(0.849)FQPS(0.176)IVMIT(0.036)IFIIIV**T**(0.996)S(0.997)QR	C	C	C	ns
Heat shock protein 90-1 (HSP90-1)	At5g52640	CY; NU	P27323	219	EI**S**(1)DDEDEDEPK	C	C	C	5.0/4.6
**PROTEIN METABOLIC PROCESS**									
Pumilio homolog 3; P1umilio homolog 1; Pumilio homolog 2	At2g29140; At2g29200; At2g29190	CY; NU	Q9ZW02; Q9ZW07; Q9ZW06	268; 272; 280	VP**S**(1)PCIT(0.999)PIGS(0.001)GR	Tr	Tr	Tr	ns
Pumilio homolog 3; Pumilio homolog 1; Pumilio homolog 2	At2g29140; At2g29200; At2g29190	CY; NU	Q9ZW02; Q9ZW07; Q9ZW06	272; 276 284	VPS(1)PCI**T**(0.999)PIGS(0.001)GR	Tr	Tr	Tr	ns
40S ribosomal protein S3-2	At3g53870	CY	Q9M339	212	TPIPDVVIIH**S**(1)PK	Tr	Tr	Tr	ns
RING/U-box domain-containing protein	At3g06330	GO	B3H497	144	I**S**(0.989)PVVS(0.004)T(0.004)QIS(0.002)AGVPGDPPNK	Tr	Tr	Tr	ns
Enhancer of mRNA-decapping protein 4	At3g13300	CY	Q9LTT8	98	TISYPTPPINIQ**S**(1)PR	4.28	4.09	4.47	ns
Eukaryotic translation initiation factor 4G (EIF4G)	At3g60240	CY	A8MR97; Q76E23	1525 1529	QVIQGPSAT(0.005)VN**S**(0.995)PR	3.45	3.37	3.53	ns
40S ribosomal proteinS2-3	At2g41840	CY	P49688	273	AI**S**(0.845)T(0.149)S(0.006)KPDPVVEDQA	2.71	2.51	2.91	ns
Serine/arginine-rich splicing factor 30 (SCL30)	At3g55460	NU^§^	Q8L3X8	204	R**S**(1)Y(0.004)S(0.996)PGYEGAAAAAPDRDR	1.84	1.26	2.43	ns
Serine/arginine-rich splicing factor 30 (SCL30)	At3g55460	NU^§^	Q8L3X8	206	RS(1)Y(0.004)**S**(0.996)PGYEGAAAAAPDRDR	1.84	1.26	2.43	ns
Putative translation initiation factor eIF-2B epsilon subunit (eIF-2B)	At2g34970	CY	O64760	447	VSIIQQPT(0.049)T(0.049)ED**S**(0.899)DEEIEY(0.003)ADSSSGTADHISGINIQMESK	C	C	C	ns
**METABOLISM**									
NADPH-cytochrome P450 reductase (CPR) 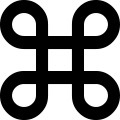	At4g24520	ER	F4JQY4	248	S(0.002)VA**T**(0.993)PYT(0.005)AVIPEYR	Tr	Tr	Tr	ns
Glutamate decarboxylase 1	At5g17330	CY; PM; GO	Q42521	8	VIS(0.064)HAV**S**(0.751)ES(0.232)DVS(0.935)VHS(0.008)T(0.007)FAS(0.003)R	Tr	Tr	Tr	ns
6-phosphofructo-2-kinase/fructose-2,6-bisphosphatase (FKFBP)	At1g07110	ER^§^; GO^§^	Q9MB58	276	SVETI**S**(1)PFQQK	2.39	2.23	2.55	ns
Photosystem Q(B) protein (psbA)	AtCg00020	PL	P83755	2	**T**(1)AIIERR	−2.57	−2.42	−2.71	na
Probable pectin methyltransferase PMT13 (QUA3)	At4g00740	GO	Q93W95	280	CIIPF**T**(0.775)AY(0.224)NAT(0.252)Y(0.749)FIEVDRIIR	−2.93	−2.04	−3.82	−2.0/ns
Probable pectin methyltransferase PMT13 (QUA3)	At4g00740	GO	Q93W95	286	CIIPFT(0.53)AY(0.468)NAT(0.037)**Y**(0.966)FIEVDRIIR	−2.93	−2.04	−3.82	−2.0/ns
Cytochrome P450, family 77, subfamily A, polypeptide 6 (CYP77A6)	At3g10570	ER	Q9SQY7	284	AIQKPGT(0.927)DKT(0.164)AS(0.154)S(0.774)F**S**(0.754)Y(0.262)IDT(0.964)IFDIK	−4.38	−4.92	−3.84	ns
Cytochrome P450, family 77, subfamily A, polypeptide 6 (CYP77A6)	At3g10570	ER	Q9SQY7	282	AIQKPGT(0.927)DKT(0.164)AS(0.154)**S**(0.774)FS(0.754)Y(0.262)IDT(0.964)IFDIK	−4.50	−4.92	−4.08	ns
ADP-glucose pyrophosphorylase family protein	At1g74910	CY	F4HXD1	217	RV**S**(0.763)S(0.237)FEAIQPATR	C	C	C	ns
**OTHER PROTEINS**									
Tetratricopeptide repeat domain protein (TSS)	At4g28080	PX	F4JKH6	1311	TSSNEISISVAGS(0.003)T(0.044)S(0.181)**S**(0.759)PAS(0.014)K	Tr	Tr	Tr	ns/−1.7
DEAD-box ATP-dependent RNA helicase 38 (LOS-4)	At3g53110	CY; NU	Q93ZG7	25	ADTVEKVPTVVES(0.005)S(0.004)S(0.007)S(0.013)S(0.011)T(0.011)VEAS(0.186)N**S**(0.762)AEK	Tr	Tr	Tr	ns
Putative bZIP protein	At3g60320	NU	Q93YU8	147	IPHIIS(0.15)ES(0.649)S(0.189)PS(0.019)S(0.08)**S**(0.912)PR	Tr	Tr	Tr	−3.0/ns
Nuclear/nucleolar GTPase (AtNug2)	At1g52980	NU	Q9C923	116	ERKIPM**S**(1)IIT(1)DNK	Tr	Tr	Tr	ns
Nuclear/nucleolar GTPase (AtNug2)	At1g52980	NU	Q9C923	119	ERKIPMS(1)II**T(**1)DNK	Tr	Tr	Tr	ns
Uncharacterized protein	At1g77765	CY	Q6DYD4	94	CDVVES(0.014)DNKPERI**S**(0.926)PS(0.059)PK	Tr	Tr	Tr	na
Uncharacterized protein	At1g19530	NU	Q93WK6	23	**S**(0.865)IT(0.135)REEIDTFWK	Tr	Tr	Tr	ns
Uncharacterized membrane protein	At3g27390	PM	Q8GUM4	544	DE**S**(0.953)IT(0.047)EPPAPVK	Tr	Tr	Tr	ns
Uncharacterized protein	At5g13260	NU	Q8VZL1	446	ISDIEIK**S**(1)PGGPK	3.65	3.11	4.20	ns
SAP domain-containing protein 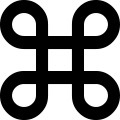	At4g39680	NU	O65655	417	VPEAQIT(0.002)NS(0.003)A**T**(0.995)PT(0.017)T(0.053)T(0.931)PR	2.91	3.20	2.62	ns
SAP domain-containing protein 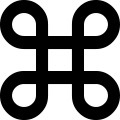	At4g39680	NU	O65655	421	VPEAQIT(0.44)NS(0.28)AT(0.28)PTT(0.001)**T**(0.999)PR	2.57	2.53	2.62	ns
Uncharacterized protein	At5g52980	CY	Q9LVV4	10	DQPDPKNG**S**(0.99)GIIIS(0.922)AT(0.088)EPIR	−1.99	−1.63	−2.35	ns
Uncharacterized protein	At5g52980	CY	Q9LVV4	15	DQPDPKNGS(0.99)GIII**S**(0.922)AT(0.088)EPIR	−1.99	−1.63	−2.35	ns
Midasin (MDN1)	At1g67120	NU	F4HRR8	4797	CG**S**(1)PQKEEPGNDIEQEPETEPIEGK	C	C	C	ns
DEK domain-containing chromatin associated protein (DEK-C)	At4g26630	CY	Q9SUA1	523	SIAHS(0.004)DDE**S**(0.996)EEEKEEEEK	C	C	C	ns
DNA topoisomerase 1 beta	At5g55310	NU	Q9FJ79	126	APS(0.002)VS(0.073)K**S**(0.9)DDEDS(0.024)EDDKPISAR	C	C	C	ns
BTB/POZ domain-containing protein	At5g67385	PM	Q66GP0	558	TSSSTIS(0.004)T(0.036)NPS(0.474)S(0.474)PIS(0.025)T(0.025)AS(0.068)**T**(0.895)GKPPIPR	C	C	C	na
Uncharacterized protein	At1g20970	PM	F4HWC3	203	NNVEEPEVEIES(0.065)DS(0.162)E**T**(0.773)DVEGHQGDK	C	C	C	ns
Uncharacterized protein 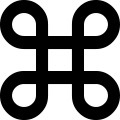	At2g15860	NU	Q8L4R9 ; F4IJD7	178; 190	IENS(0.005)VQQGS(0.101)**S**(0.894)PR	C	C	C	ns
Uncharacterized protein	At4g31880	NU	F4JTF2; Q8GUP3	558; 559	AIDEES(0.002)IHT(0.053)**S**(0.88)S(0.064)GDNEKPAVSSGK	C	C	C	ns
Uncharacterized protein	At5g64090	PL	Q9FMI8	8	MDFS(0.034)VKP**S**(0.772)GGS(0.159)PS(0.034)PSSSTSSSTPHR	C	C	C	1.6/ns

a Phosphosites in each functional category are listed according to the average log2 ratio (Treated/Control), in descending order.

b Full name of the identified protein. Proteins that have never been described before as phosphorylated are underlined. 
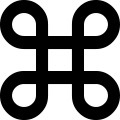
 indicates proteins that have been identified as regulated by OGs in Kohorn et al. (2016).

c ID of the identified protein from the TAIR database (The Arabidopsis Information Resource database. www.arabidopsis.org)

d Subcellular localization, obtained from SUBA (the SUBcellular localization database for Arabidopsis proteins, http://suba3.plantenergy.uwa.edu.au/). PL, plastid; EX, extracellular; CY, cytosol; PM, plasma membrane; ER, endoplasmic reticulum; GO, Golgi apparatus; VO, vacuole; NU, nucleus; PX, peroxisome; MI, mitochondrion; CS, cytoskeleton. ^§^indicates localization determined by GFP fusion.

e ID of the identified protein, from the UniProtKB database (www.uniprot.org/). Proteins that have never been described before as phosphorylated at the residues reported here are ud.

f Position of the phosphorylated amino acid on the whole protein sequence.

g Phosphopeptide sequence with the localization probability score assigned to each site and the phosphorylated amino acid (bold and ud).

h Average fold change (log2) between corresponding OG-treated and control phosphosites (Tr/C). Positive and negative values indicate increases and decreases in phosphorylation, respectively, upon treatment. C indicates proteins that are exclusively present in control samples and that are not detected in all replicates of OG- treated samples. Tr indicates proteins that are exclusively present in OG-treated samples and that are not detected in all replicates of control samples.

i Fold change (log2) between corresponding OG-treated and control phosphosites (Tr/C) in each replicate.

j Fold change of transcript levels after OG treatment at 1 and 3 h with respect to the mock-treated control (Denoux et al., 2008). Only data of genes for which fold change is significant and ≥1.5 in at least one time-point are shown. ns, not significant (P > 0.01); na, not applicable.

Proteomic analysis of the TMF did not show substantial changes in overall protein abundance (Data Sheet S2B in Supplementary Material). This is expected, as a significant *de novo* protein synthesis or protein degradation is unlikely to occur within 10 min of elicitor treatment (Benschop et al., 2007). Identified proteins (Data Sheet S2A in Supplementary Material) were classified according to known gene ontology using AgriGO (http://bioinfo.cau.edu.cn/agriGO/analysis.php). GO term analysis of the cellular component of the total list of proteins demonstrated a clear enrichment for PM-associated proteins, cell wall and intracellular organelles (Figure 1C).

### Identification of OG-regulated phosphoproteins by phospho-DIGE

**Figure 2:**
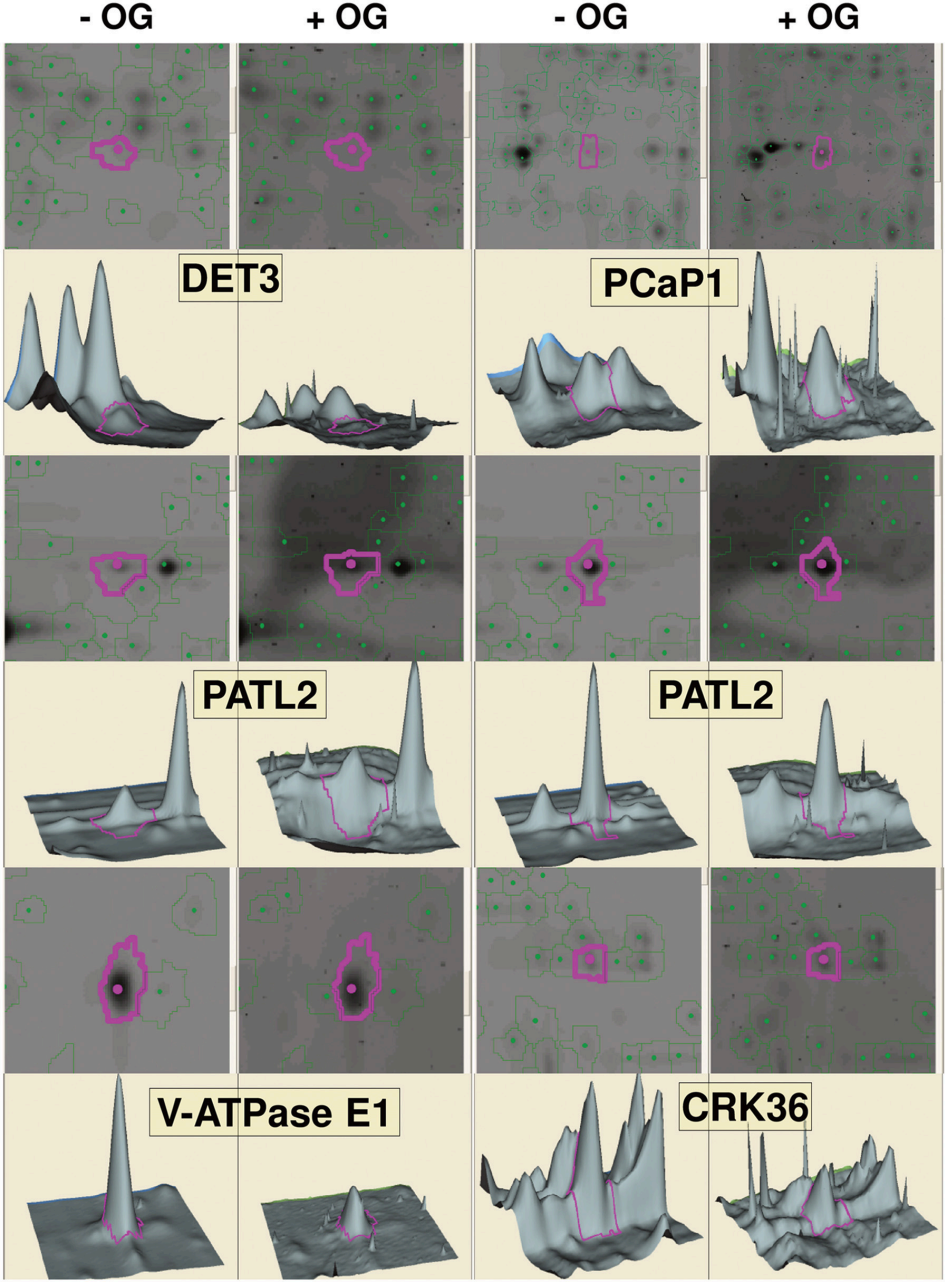
**Gel visualization of selected spots from DeCyder analysis**. Representative images showing differentially phosphorylated spots upon OG treatment for each protein, gel images of the spots, along with the corresponding 3D images to visualize the phosphorylation changes, are indicated by purple circles. Two spots are shown for PATL2; one (left) exhibits increased phosphorylation in response to OGs, while the other one (right) shows a slight but significant dephosphorylation (−1.15-fold; see Table 2).

In a second approach, phospho-DIGE was exploited for detecting changes in protein phosphorylation using both TMFs and total protein extracts. Three independent biological replicates were performed for each treatment and samples were labeled according to the randomization scheme shown in Table S3, to enforce statistical analysis. About 800 fluorescent spots were detected for the total extracts, and about 500 spots for the TMFs. Statistical analysis revealed 30 spots in the total extract and 50 spots in the TMF fraction that changed their phosphorylation status (*p* < 0.05) after treatment with OGs (representative gel images are shown in Figures S1, S2). The spots were analyzed by MALDI-TOF-MS, leading to the identification of 46 differential phospho-isoforms, corresponding to 34 phosphoproteins (Table 2). Representative images showing spots that exhibited an increase or decrease in phosphorylation upon OG treatment, along with representative 3D images to visualize the phosphorylation changes, are shown in Figure 2. Proteomic analysis showed that, again, differences in non-phosphorylated protein abundance between OG-treated and control samples were not significant, demonstrating that most changes occurred at the PTM level.

**Table 2:** **List of 2D-DIGE identified phosphoproteins regulated by OGs**.

**Protein name^a^**	**TAIR ID^b^**	**Loc^c^**	**Spot^d^**	**Score^e^**	**Cov^f^**	**No. of peptides^g^**	**ANOVA^h^**	**Fold change^i^**	**μarray OG 1 h/3 h^j^**
**TRANSPORT**									
ATPase subunit beta-1 (ATPase b1)	At5g08670	MI	R	76 (32)	20	9	0.0049	−1.40	ns
Vacuolar ATP synthase subunit C (VHA-C)/ De-Etiolated 3 (DET3)	At1g12840	VO GO PM	Y	60 (36)	28	10	0.00045	−1.91	ns
Vacuolar ATP synthase subunit A (VHA-A)	At1g78900	VO GO	C	241 (346)	42	27	0.0050	−2.28	ns
Vacuolar ATP synthase subunit E1 (VHA-E1)	At4g11150	VO GO	1S	123 (25)	46	15	0.0026	−2.98	na
**RECEPTORS, KINASES, PHOSPHATASES**									
Toll-Interleukin-Resistance domain-containing protein	At1g72910	CY	S	60 (18)	23	4	0.0026	1.65	3.2/1.9
PKS2 (phytochrome kinase substrate 2)	At1g14280	CY NU	1R	72 (38)	15	7	0.0012	1.44	−3.1/ns
MCK 7.17 LRR Protein Kinase	At5g58300	PM	12^*^	190 (78)	12	6	0.0015	1.41	ns
Calcineurin B-like protein 9 (CBL9)	At5g47100	PM	1W	42 (68)	24	6	0.0073	1.36	ns
Protein phosphatase 2C-like (P2C17)	At1g78200	PM	1H	661 (282)	12	6	0.0046	−1.27	ns
Cysteine-rich receptor-like protein kinase 36 (CRK36)	At4g04490	PM	L	441 (10)	15	6	0.00055	−2.16	3.7/ns
**MEMBRANE TRAFFICKING**									
Patellin2 (PATL2)	At1g22530	PM	D	81 (16)	28	13	0.0028	2.27	−2.0/−1.6
			E	64 (16)	14	8	0.020	−1.15	
Actin 7 (ACT7)	At5g09810	NU MI PM	X	136 (107)	49	15	0.0012	1.95	ns
PCaP1/MDP25	At4g20260	PM^§^	1I	160 (38)	71	14	0.0010	1.85	−1.7/ns
			1P	98 (40)	33	16	0.00013	1.83	
TUA5 (tubulin alpha-5)	At5g19780	CY^§^	V	132 (45)	25	25	0.0066	1.19	ns
**RESPONSE TO STRESS**									
Glutathione S-Transferase (GST-PM24)	At4g02520	CY^§^	1T	138 (143)	66	12	0.0019	1.60	2.0/2.8
Luminal-binding protein 1 (BiP 1)	At5g28540	ER^§^	A	65 (58)	14	9	0.0019	1.49	ns/2.7
Glutathione S-Transferase (GSTF8)	At2g47730	PL CY^§^	1V	52 (33)	15	4	0.0046	1.43	2.2/2.2
Glutathione S-Transferase (GSTF9)	At2g30860	CY^§^	1X	54 (63)	26	6	0.0096	1.35	ns/1.8
CLPC1 (heat shock protein 93-V) HSP93-V	At5g50920	PL	6^*^	233 (71)	17	9	0.033	1.32	ns
			7^*^	18 (90)	15	6	0.014	1.39	
Heat Shock Protein 70-1 (HSP70-1)	At4g24280	PL	2^*^	96 (31)	21	14	0.04	1.26	ns
			4^*^	85 (22)	17	8	0.006	1.27	
Flavodoxin-like quinone reductase 1 (FQR1)	At5g54500	PM	1U	75 (68)	50	8	0.0021	1.20	1.8/ns
Heat Shock Cognate 70-1 (HSC70-1)	At5g02500	CY	13^*^	85 (66)	16	7	0.05	1.19	ns
CPN60B (Chaperonin 60 Beta)	At1g55490	PL	14^*^	94 (79)	19	6	0.014	1.13	ns
Monodehydroascorbate reductase 2 (MDAR2)	At5g03630	CY	W	98 (22)	37	12	0.0028	−1.61	2.5/2.2
**JACALIN LECTIN-LIKE PROTEINS**									
PYK10	At3g09260	ER^§^	19^*^	84 (38)	15	8	0.034	−1.15	ns
JAL34	At3g16460	CY PX^§^	5^*^	171 (45)	20	7	0.041	−1.2	ns
JAL27	At3g16390	EX CY	25^*^	33 (17)	25	6	0.044	−1.47	ns
**METABOLIC PROCESS**									
Glyceraldehyde-3-phosphate dehydrogenase (GAPC1)	At3g04120	CY^§^	1G	571 (101)	29	9	0.00043	2.25	ns
			1E	601 (159)	27	6	0.038	2.09	
			1D	791 (56)	37	11	0.0024	1.39	
			1F	581 (91)	23	7	0.0035	1.16	
NADP-malic enzyme 2 (NADP-ME2)	At5g11670	CY	N	61 (16)	13	7	0.0038	1.84	3.8/2.9
Methionine Synthase (MS1)	At5g17920	CY^§^	O	74 (13)	14	9	0.0020	1.82	ns
			17^*^	94 (36)	18	11	0.037	1.32	
			18^*^	127 (35)	20	15	0.017	1.27	
			16^*^	83 (98)	19	13	0.042	1.26	
			15^*^	84 (87)	9	6	0.0042	1.09	
Major latex protein-related	At4g23670	CY VO	2Z	63 (70)	24	6	0.001	1.56	ns
CICDH, isocitrate dehydrogenase (Cicdh)	At1g65930	CY	U	119 (33)	24	11	0.0048	1.54	ns
Triosephosphate isomerase, cytosolic (CTIMC)	At3g55440	CY	24^*^	127 (62)	43	10	0.016	−1.69	ns
60S acidic ribosomal protein P0 (60S-P0)	At3g09200	CY	1O	62 (32)	18	3	0.0015	−1.81	ns
			1J	72 (29)	18	16	0.00025	−2.66	

a Full name of the identified protein. Proteins that have never been described before as phosphorylated are ud.

b ID of the identified protein from the TAIR database (The Arabidopsis Information Resource database. www.arabidopsis.org).

c Subcellular localization, obtained from SUBA (the SUBcellular localization database for Arabidopsis proteins, http://suba3.plantenergy.uwa.edu.au/). PL, plastid; EX, extracellular; CY, cytosol; PM, plasma membrane; ER, endoplasmic reticulum; GO, Golgi apparatus; VO, vacuole; NU, nucleus; PX, peroxisome; MI, mitochondrion; CS, cytoskeleton. ^§^indicates localization determined by GFP fusion.

d Numbers correspond to spots shown in Figure S1 (total protein extracts, indicated by ^*^) or in Figure S2 (total microsomal fraction).

e *Value obtained from MASCOT (http://www.matrixscience.com) reported as a measure of the statistical significance of a match (>60). In parenthesis are shown the values obtained from ALDENTE (ftp.expasy.ch/tools/aldente) independently (significance> 13.86)*.

f Percentage of protein sequence covered by identified peptides.

g Numbers of different identified peptides.

h ANOVA: the Student's t-test p-value represents the probability of obtaining the observed ratio if control and OG-treated spots have the same protein abundance. Significant values (p < 0.05) are reported.

i Fold change is calculated as the ratio of the average standardized abundances corresponding to OG-treated and control spots after ProQ Diamond staining. Positive and negative values are indicated for increases and decreases in phosphorylation state, respectively, upon treatment.

j Fold change of transcript levels after OG treatment at 1 and 3 h with respect to the mock-treated control (Denoux et al., 2008),. Only data of genes for which fold change is ≥1.5 and significant in at least one treatment are reported. ns, not significant (P > 0.01). na, not applicable.

### Computational analysis of the differentially phosphorylated proteins

To summarize, LC-MS/MS analysis of TMF led to identification of 58 and 42 sites exhibiting an increased or decreased phosphorylation, respectively (Table 1), whereas Phospho-DIGE analysis of both TMF and total extracts revealed a total of 32 and 14 phosphoprotein spots showing an increased or decreased phosphorylation, respectively, corresponding to 34 proteins due to the presence of multispots (Table 2). Only Patellin 2 (PATL2) was found to be differentially phosphorylated in both analyses of the TMF. Two spots are shown for PATL2 (Figure 2 and Figure S2); one exhibits increased phosphorylation in response to OGs, in agreement with our detection of a phosphopeptide exclusively present in the OG-treated sample (Table 1). The other PATL2 spot shown in Figure 2 shows a slight but significant dephosphorylation (−1.15-fold). Many studies reported comparative analyses using both gel-based and gel-free methods, where the match of identified proteins is very low, and emphasized that these two strategies are indeed complementary (Kleffmann et al., 2007; Zhao et al., 2008; Robbins et al., 2013).

**Figure 3:**
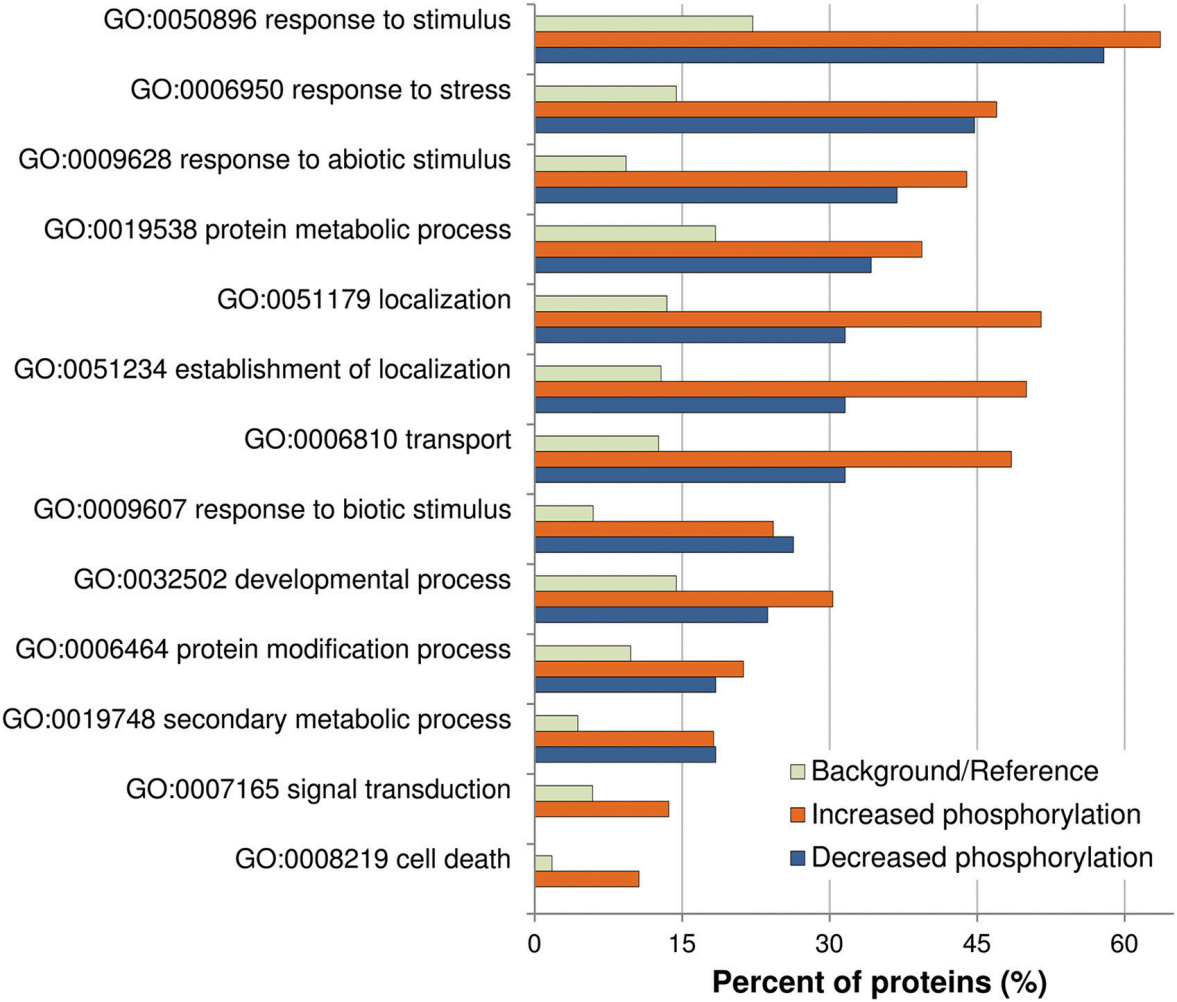
**Analysis of enriched GO term distributions within the differentially phosphorylated proteins**. The analysis was performed on the differentially phosphorylated proteins listed in Table 1 (LC-MS/MS) and Table 2 (Phospho-DIGE) using the GO annotations of biological process in the AgriGO database (Y axis). Barplot shows selected GO terms significantly enriched (FDR < 0.05, Yekutieli adjusted) for proteins with increased or decreased phosphorylation. The background/reference represents the proportion of all annotated genes of each GO term within the total genes in the TAIR10 database.

Previously described phosphorylation sites were searched using the PhosPhAt (http://phosphat.mpimp-golm.mpg.de/) and P3DB (http://www.p3db.org/) databases. Among all the differential phosphoproteins, sixteen [underlined in Table 1 (9 proteins) and Table 2 (7 proteins)] have never been described to be phosphorylated at the identified sites. GO term enrichment analysis with AgriGO was performed to identify significantly over-represented biological process categories in the subset (Tables 1, 2) of OG-regulated phosphoproteins (Figure 3). The GO terms referring to response to various biotic and abiotic stimuli were significantly enriched in the analyzed subsets of differential phosphoproteins, showing that the molecular processes involved in the response to OGs indeed correlate with a condition of stress. Proteins with increased phosphorylation were specifically enriched in terms referring to Signal transduction and to Cell death, while decreased phosphorylation occurred mainly in proteins associated with Response to biotic stimulus (Figure 3).

**Figure 4:**
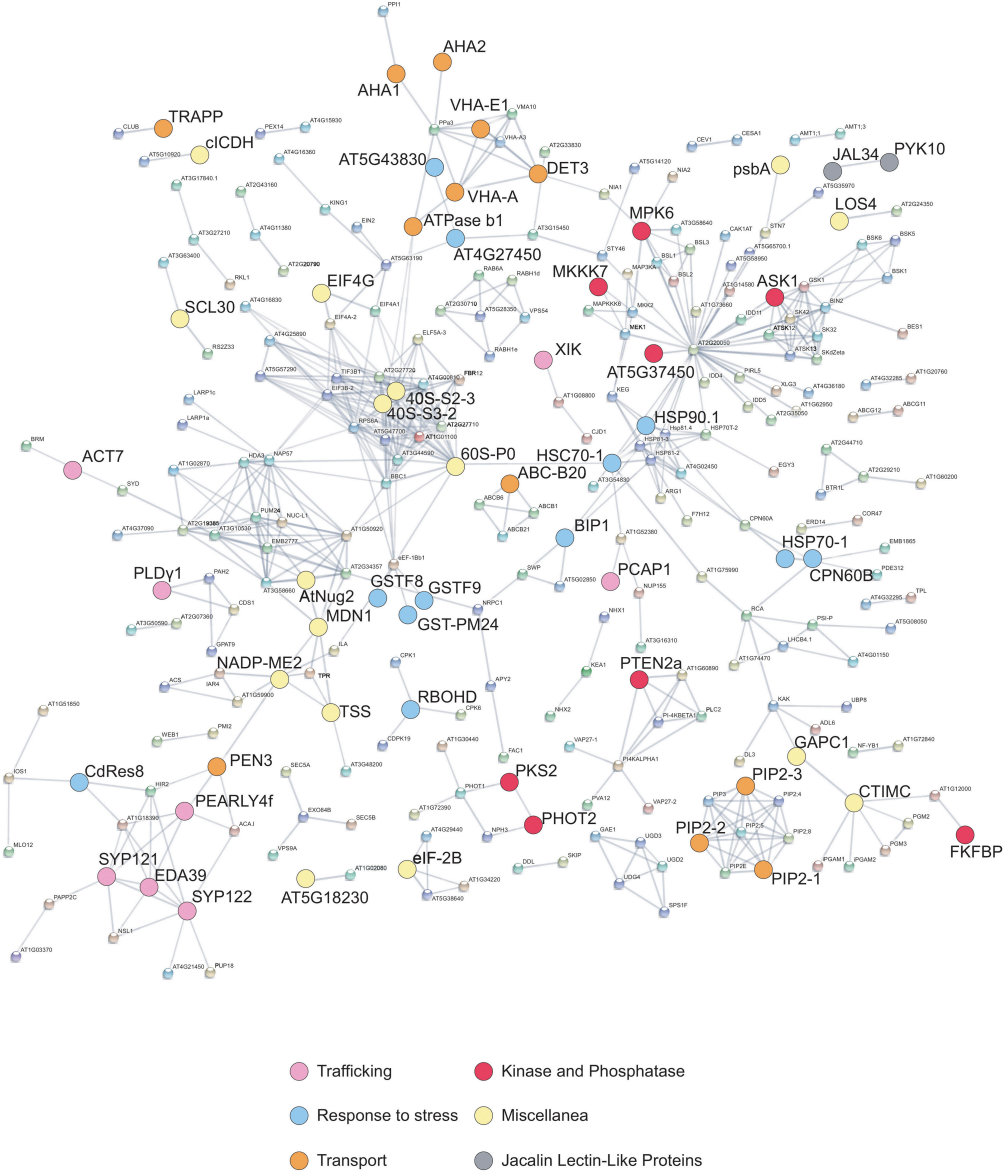
**STRING interaction network**. The graph shows association between proteins corresponding to all phosphosites quantified by LC-MS/MS analysis (listed in Data Sheet S1B in Supplementary Material) as well as differentially phosphorylated proteins identified by Phospho DIGE analysis (Table 2). The interaction map was generated using high- confidence (>0.7) criteria for linkage, taking into account co-expression, experimental evidences, and existing databases. Differentially phosphorylated proteins listed in Tables 1, 2 are highlighted and color coded based on functional categories.

Connections between all the quantified phosphorylated proteins were searched by using the STRING algorithm (Figure 4). Proteins with differentially regulated phosphosites are highlighted with colors corresponding to different functional categories. Many clusters were identified, with several interesting networks standing out. The highly interconnected “transport” cluster includes several primary electrogenic proton pumps found in all eukaryotes: the PM H^+^-ATPases AHA1 and AHA2, different subunits of the vacuolar H^+^-ATPase (V-ATPase or VHA; subunits A, C, and E1; Batelli et al., 2007) as well as the subunit beta-1 of the mitochondrial ATP synthase. The V-ATPase subunit C, encoded by the single gene *DE-ETIOLATED3* (*DET3)*, is directly connected with the other V-ATPase subunit E1, but also with AHA1 and AHA2.

Multiple interactions are evident among elements involved in signaling (Figure 4), such as kinases, receptor kinases, and phosphatases. Many kinases appear to be connected to PP2C-type phosphatase-like protein (At2g20050), an enzyme that acts as negative modulator of protein kinase pathways involved in stress and developmental processes (Kuhn et al., 2006). Moreover, an additional smaller cluster includes several heat-shock proteins (HSP90-1, HSP70-1, HSP93-V, HSC70-1, BiP1) that are connected also to ribosomal proteins (Figure 4). Proteins involved in intracellular trafficking, namely syntaxin SYP 121 and 122, a calmodulin-binding protein (EDA39), PCaP1 (Plasma membrane-associated cation-binding protein 1), and a phospholipase like protein (PEARLI 4f) form an additional cluster.

## Discussion

Previous proteomic studies have either postulated (Chivasa et al., 2006; Nuhse et al., 2007) or shown (Jones et al., 2006) that PTM-mediated regulation is important for plant immunity. Here we have studied very early protein dynamics induced by OGs, an important class of DAMPs, employing two complementary proteomic approaches, SIMAC phosphopeptide enrichment followed by LC-MS/MS and Phospho-DIGE (a gel-based approach). These analyses led to the identification of 100 regulated phosphosites and 46 differentially phosphorylated protein spots, respectively (Tables 1, 2). The functional classification of the OG-regulated phosphoproteins, based on the GO functional categories, identifies typical major PM functions. Three main categories emerged: transporter proteins, signaling proteins (receptors, kinases, phosphatases) and proteins involved in membrane trafficking.

A comparison of the OG-regulated phosphoproteome identified in this study with that regulated by flg22, which includes 127 phosphoproteins identified in three large-scale proteomics works (Nuhse et al., 2007; Benschop et al., 2007; Rayapuram et al., 2014), shows only 18 shared phosphosites, corresponding to 14 phosphoproteins (Table S4). Among these, there are MPK6, which shows OG-induced phosphorylation at the same residues that undergo flg22-induced phosphorylation (i.e., Thr221 and Tyr223), RBOHD and Penetration3 (PEN3), an ATP-binding cassette (ABC) family 36 transporter that undergoes phosphorylation also in response to H_2_O_2_ and methyl jasmonate (Stecker et al., 2014). ABC transporters are integral membrane proteins that transport a wide variety of substrates, such as ABA, auxin, and some plant secondary metabolites across cellular membranes (Kuromori et al., 2010).

Interestingly, twenty-two of the OG-regulated phosphoproteins identified here are reported as targets of MPK3 and MPK6 (Table S5; Lee et al., 2015). Among these, the putative tyrosine phosphatase PTEN2a shows OG-dependent increased phosphorylation. This enzyme has been shown to dephosphorylate also the 3′ phosphate group of PI3P (phosphatidylinositol 3-phosphate) *in vitro* and to possess strong binding affinity for PA (phosphatidic acid), an important second messenger with roles in stress and hormonal signaling (Pribat et al., 2012). Lipid signaling pathways activated downstream of the MAPKs may therefore be involved in the response to OGs. A phosphotyrosine site that disappears in response to OGs and may be a target of PTEN2a is present in the GSK/Shaggy-like kinase/ASK1 at position 229. Auto-phosphorylation of this residue is required for ASK1 trans-phosphorylation activity (de la Fuente van Bentem et al., 2008) and activation of the cytosolic glucose-6-phosphate dehydrogenase (G6PD), which is essential for maintaining the cellular redox balance (Dal Santo et al., 2012). ASK1 dephosphorylation may thus lead to reduced G6PD activity and elevated ROS levels that contribute to the elicitor-induced oxidative burst.

MPK3 and MPK6 activation promotes also the biosynthesis of indole glucosinolates and the conversion of indole-3-yl-methylglucosinolate (I3G) to 4-methoxyindole-3-yl-methylglucosinolate (4MI3G), through the phosphorylation of ETHYLENE RESPONSE FACTOR 6 (ERF6). 4MI3G is a substrate of the atypical myrosinases PEN2 and PEN3, which shows increased phosphorylation in response to OGs, and is converted to extracellular unstable anti-microbial compounds, possibly isothiocyanates (Xu et al., 2016). OGs may therefore induce production of indole glucosinolates, but this aspect has been scarcely investigated so far.

A few differentially phosphorylated proteins identified here show regulation also at the level of gene expression, with more than a 1.5-fold change in the corresponding transcript levels within 3 h (Tables 1, 2; Denoux et al., 2008), pointing to a regulation at two different levels. Most of the identified proteins, however, show mainly a PTM-based regulation, by phosphorylation/dephosphorylation, suggesting that only activation/inactivation or stabilization/degradation, but not *de novo* protein synthesis, is necessary to exert their physiological role.

### Signal transduction proteins

Several identified proteins are likely players in signaling. Among the proteins involved in regulation through phosphorylation/dephosphorylation, we found members of the Receptor-Like Kinases (RLK) family, including members of the Leucine-Rich Repeat RLK (LRR-RLK) sub-family, cyclin-dependent protein kinases, PTEN2a, and the MAP triple kinase MKKK7 (At3g13530, also known as MAP3Kepsilon 1). MKKK7 has been described to interact with FLS2 and to be phosphorylated at Ser452/Ser854 in response to flg22, functioning as a negative regulator of flg22-triggered MPK6 activation (Mithoe et al., 2016). We find, instead, dephosphorylation of MKKK7 on Ser788 after OG perception, suggesting a distinct event in the signaling cascades initiated by the two elicitors. Another immune-related protein, the Cysteine-rich Receptor-like protein Kinase 36 (CRK36), exhibits decreased OG-dependent phosphorylation, along with up-regulation at the transcript level. CRK36 interacts with FLS2 and its overexpression leads to enhanced PTI and resistance to virulent bacteria *Pseudomonas syringae* pv. tomato DC3000, constitutive accumulation of callose and constitutive stomatal closure (Yeh et al., 2015). A role of CRK36 in resistance to insects has also been proposed (Barah et al., 2013). Moreover, *CRK36* silencing leads to higher sensitivity to ABA and osmotic stress during the post-germination growth phase, suggesting that the protein negatively controls ABA signaling (Tanaka et al., 2012).

We also identified three phospholipases (PLDγ1 and two putative phospholipases, pEARLI4, and pEARLI4-f) as well as CBL9, a myristoylated PM-localized protein that does not possess enzymatic activity and contains four EF hands (Batistic et al., 2008). CBL9 modulates ABA responses (Kim et al., 2003) and forms complexes with CBL-interacting protein kinases (CIPKs), leading to phosphorylation of AHA2 (Fuglsang et al., 1999) and regulation of V-ATPases (Tang et al., 2012), also found among the OG-regulated phosphoproteins. CBL-CIPK complexes act as a two-module Ca^2+−^-decoding system where CBL phosphorylation is required for CIPK-mediated phosphorylation of target proteins (Steinhorst and Kudla, 2013) and regulation of channel activity, ion fluxes and ROS formation during environmental adaptation reactions (Kurusu et al., 2015).

It is worth noting that remorin REM1.3, identified as phosphorylated on Thr58 in response to both OGs and flg22 (Kohorn et al., 2016; Benschop et al., 2007), was also identified in our analysis as phosphorylated at the same site, but with a Significance B *p*-value of 0.1. A tomato remorin was the first protein described to bind and to be phosphorylated in response to OGs (Reymond et al., 1996) and potato REM1.3 has been shown to accumulate in discrete peri-haustorial domains and enhance susceptibility to *Phytophthora infestans* (Bozkurt et al., 2014). Arabidopsis REM1.3 is differentially recruited to detergent-insoluble membranes/detergent-resistant membranes (DIMs or DRMs), a term that indicates lipids and proteins that have been biochemically associated with sphingolipid (SL)- and sterol-enriched membranes (Tapken and Murphy, 2015).

Two proteins functioning in the transduction of the light signal, PHOT2 and Phytochrome Kinase Substrate 2 (PKS2), exhibit increased phosphorylation in response to OGs. *pks2* loss-of-function mutants show altered expression patterns of auxin marker genes (Kami et al., 2014) and it is tempting to speculate that PKS2 is involved in auxin OG/antagonism, the molecular basis of which is still unknown (Savatin et al., 2011). Notably, the auxin-response factor ARF1, a repressor ARF (Chandler, 2016), showed decreased phosphorylation upon OG treatment at two novel phophosites, the adjacent residues Ser399 and Ser400 located in middle region of the protein thought to function as the repression domain (Ulmasov et al., 1999). Ser405, close to this novel dual phosphorylation site, has been described as phosphorylated in seedlings (Wang et al., 2013) but does not appear to be, regulated by OGs. Very little is known about how phosphorylation controls ARF activity. ARF2, also a repressor ARF that is phylogenetically sister to ARF1, loses its DNA-binding capability upon phosphorylation by the brassinosteroid-regulated kinase BIN2 (BRASSINOSTEROID-INSENSITIVE2), providing an example of hormonal crosstalk at the post-translational level (Vert et al., 2008). On the other hand, phosphorylation of the activator ARFs ARF7 and ARF19 increases their DNA binding ability and enhances transcription (Cho et al., 2014). In the light of the OG-auxin antagonism, OG-induced phosphorylation of ARF1 may lead to its activation as a repressor.

### Trafficking-related proteins

OGs induce increased phosphorylation of cytoskeleton-related phosphoproteins such as myosin-17 (XIK), Actin7 (ACT7), and PCaP1. Myosin XIK is required for normal movements of actin filaments (Park and Nebenfuhr, 2013), turnover of actin and trafficking of vesicles carrying non-cellulosic cell wall components (Cai et al., 2014). The protein acts as a key regulator of plant antifungal immunity and contributes to cell wall-mediated penetration resistance, including deposition of callose and accumulation of PENETRATION1 (PEN1)/SYP121 and lignin-like compounds at the penetration site, when highly expressed (Yang et al., 2014). Pharmacological inhibition of myosins affects accumulation of PEN3 more than that of PEN1, suggesting that transport pathways mediating recruitment and export of the two proteins to apoplastic papillae are distinct (Underwood and Somerville, 2013). OG-induced phosphorylation of myosin XIK may regulate the motility of secretory vesicles and PEN recruitment.

ACT7 is one of the three vegetative and partially redundant isoforms of the actin family and plays a role in germination and root growth (Gilliland et al., 2003; Kijima et al., 2015). Expression of *ACT7* strongly responds to auxin and wounding and is required for callus formation (Kandasamy et al., 2001); as shown in Figure 4, it is also coordinated with the expression of *GAPC1* (Glyceraldehyde-3-phosphate dehydrogenase), the product of which also shows increased phosphorylation in response to OGs. How phosphorylation regulates actin function in plants is not known, except for *Mimosa pudica* actin, which is heavily tyrosine-phosphorylated, and dephosphorylated at different extent depending on the degree of bending of the petiols (Kameyama et al., 2000).

PCaP1 is capable of binding actin and calcium, leading to the destabilization of actin filaments, besides phosphatidylinositol phosphates (Qin et al., 2014). It is localized *via* N-myristoylation on the PM, from which it dissociates at increased Ca^2+^ levels (Li et al., 2011). The protein negatively regulates hypocotyl (Li et al., 2011) and pollen growth (Qin et al., 2014); moreover, it is induced by brassinosteroids and its overexpression partially rescues the morphological phenotype of the *det2* mutant, defective in BR biosynthesis (Tang et al., 2008). PCap1 has never been implicated in immunity.

Components of the microtubule (MT) apparatus, namely TUBULIN ALPHA-5 (TUA5) and the 65-kDa MT-associated protein 1 (MAP65-1), a structural element involved in MT bundling and cell division, also show OG-regulated increase in phosphorylation. MTs are disrupted or rearranged in response to elicitors and stable MTs negatively affect defense (Guan et al., 2013). Phosphorylation of MAP65-1 was found exclusively in OG-treated samples, at residues located within the second MT-interacting region. Phosphorylation of this region has been shown to diminish the interaction with MTs, also in tobacco (Guan et al., 2013) and involve CDPKs and MAPKs (Smertenko et al., 2006), which are known to participate in OG signaling cascade (Ferrari et al., 2013; Gravino et al., 2015).

Vesicle trafficking appears to be another important target of the OG regulatory action. SYP122 decreases phosphorylation in response to OGs, while SYP121/PEN1 (Reichardt et al., 2011) as well as PATL2, PATL3, and PATL4 show an opposite behavior. PATL1 is essential for cell plate formation and maturation at late telophase, when dynamic vesicle trafficking and vesicle fusion occur (Peterman et al., 2004); PATL2 is related to the phosphatydylinositol transfer protein Sec14, which plays a role in the interplay between lipid metabolism and membrane trafficking, whereas PATL3 has been shown to interact with the movement protein of Alfalfa Mosaic Virus and interfere with viral movement (Peiro et al., 2014).

A phosphosite of the kinesin motor protein KAC1 (At5g10470) was found only in the presence of OGs. KAC1 is phoshorylated also in response to flg22 (Table S4) and xylanase treatment (Benschop et al., 2007). Kinesins function in vesicle transport along MTs and their phosphorylation is thought to be required for correct folding and dimerization. The C-terminal domain of KAC1 and KAC2, interact with F-actin *in vitro* and mediate chloroplast movement (Suetsugu et al., 2010). Both proteins belong to the kinesin-14 subfamily (Zhu and Dixit, 2012), greatly expanded in land plants and representing the largest Arabidopsis kinesin subfamily (21 members). Expansion is attributed to the lack of the MT-based motor protein dynein in plants, and some of the kinesin-14 subfamily members may substitute for dynein to perform retrograde transport functions (Muller, 2015).

### ATPases and other membrane transporters

Initial pathogen recognition occurs at the PM and many of the early responses involve membrane transport processes. For example, activity of PM AHAs is dynamically regulated during plant immune responses and multiple pathogens target this family of enzymes. AHAs are responsible for creating and maintaining a negative membrane potential and a transmembrane pH gradient (acidic outside) that control multiple aspects of transport across the PM. Regulatory phosphorylation sites identified in AHAs are located either in the N- or C-terminal domain (Rudashevskaya et al., 2012). Phosphorylation of the penultimate residue, a Thr, within the auto-inhibitory C-terminal domain is critical for interaction with a 14-3-3 regulatory protein (Fuglsang et al., 1999); moreover, phosphorylation within this domain affects enzyme activity incrementally, suggesting a fine modulation (Speth et al., 2010). OGs induce phosphorylation of AHA1 and AHA2 at the conserved residue Ser899, a modification that has been reported to inhibit proton transport (Haruta et al., 2014) and to occur in response to flg22 (Nuhse et al., 2007) This modification may play a role in the extracellular alkalinization induced by elicitors, including OGs. Moreover, OGs induce phosphorylation of AHA1 at the non-conserved Ser544, located in the central part of the protein. Phosphorylation at this site has been recently described (Rudashevskaya et al., 2012) but, to our knowledge, never in response to elicitors.

Several subunits of V-ATPase (VHA-A, VHA-E1, and DET3) also change their phosphorylation status in response to OGs. V-ATPases are multi-subunit enzymes resembling the procariotic ATPases and therefore performing rotational catalysis. V-ATPases are localized on both the tonoplast and the Trans-Golgi Network (TGN) and their impairement affects morphology and function of the Golgi and TGN (Schumacher and Krebs, 2010). In both plants and animals (Robinson, 2014), V-ATPase perform uphill transport of protons and do not only energize secondary active transport, but also play a relevant role in vesicle trafficking and post-translational processing of secretory proteins, by regulating endosomal pH (Huotari and Helenius, 2011). Micro-compartments along the endocytic and secretory pathways have characteristic luminal pH values suited to their functions, and specific luminal pH and ionic concentration may be required not only for appropriate enzyme activities but also for membrane trafficking and sorting and cargo routing. Progressively decreasing pH in the endocytic pathway are thought to provide the cargo information about its location within the pathway (Huotari and Helenius, 2011; Martiniere et al., 2013).

OGs regulates phosphorylation of other plasma membrane transporters such as the acquaporin PIP2-1 and, besides PEN3, two additional ABC transporters, At3g55320 and At3g62700, belonging to subfamily 20 and 14, respectively. Ser280, Thr712 and Ser925 in PIP2-1, At3g55320, and At3g62700, respectively, showed phosphorylation only in the presence of OGs. In PIP2-1, phosphorylation at Ser280 and Ser283, has been associated to the regulation of hydraulic conductivity (Prado et al., 2013). Moreover, the latter phosphosites is necessary for correct targeting to the PM (Prak et al., 2008), showing that specific phosphorylation events control not only activity, but also targeting of transporters. Interestingly, PIP2-1 along with some members of family B and G ABC transporters not identified in this study, are markers of the so-called membrane nanodomains (NDs), ordered membrane SL- and sterol-rich microenvironments with functional assemblies of lipids and proteins that are defined through biophysical or microscopy, and not biochemical, techniques (Tapken and Murphy, 2015), and therefore are distinct from DIMs/DRMs, where PEN3 is instead found.

### Response to stress

A small cluster of OG-regulated phosphoproteins is represented by heat-shock proteins, including the cytosolic heat-shock cognate 70-1(HSC70-1), the chloroplast heat shock protein 70-1 (HSP70-1), BiP1 and the cytosolic HSP90. HsP90 is a molecular chaperone responsible for the maturation and stability of a large number of signaling proteins, in particular protein kinases. We found decreased levels of the HSP90 Ser219 phosphosite, in agreement with previous reports (Reiland et al., 2009; Jones et al., 2009). The role of this modification is not known. In yeast, key phosphorylation sites on HSP90 affect the interaction with a selected subset of co-chaperones, and consequently, client proteins (Mollapour et al., 2011); a similar role is possible in plants. Arabidopsis HSP90 interacts with MPK4 and regulates its kinase activity during response to flg22 (Cui et al., 2010). HSP90 isoforms, together with the co-chaperones RAR1 and SGT1, appear to be critical also for the stability of nucleotide-binding leucine-rich repeat receptor (NLR) resistance proteins such as RPM1, MLA, RPS2, RPS4, I2, and N, and the formation of immune receptor complexes (Huang et al., 2014). In addition, they negatively regulate the accumulation of NLRs, likely to avoid autoimmunity (Huang et al., 2014). The decreased level of phosphorylation in response to OGs is likely related to the regulation of HSP90 complex assembly and substrate specificity.

### Proteins involved in redox homeostasis

Many proteins involved in redox homeostasis and ROS signaling show OG-induced increase in phosphorylation, including an isocitrate dehydrogenase (CICDH; Table 2) involved in response to pathogens (Mhamdi et al., 2010), and GAPC1, a glycolytic enzyme that interacts with phospholipase D and transduces hydrogen peroxide signals during stress (Guo et al., 2012). Both proteins appear to be regulated mainly through PTM. In fact, only few proteins involved in redox regulation here identified show both phosphorylation changes and transcript up-regulation. Among these, RBOHD (Kadota et al., 2014), glutathione S-transferases (GSTF9, GST-PM24, GSTF8), the non-photosynthetic NADP-malic enzyme 2 (NADP-ME2) and flavodoxin-like quinone reductase 1 (FQR1) exhibit increased phosphorylation, whereas a monodehydroascorbate reductase (MDAR2) and the protein plant cadmium resistance 8 (CdRes8) show decreased phosphorylation. NADP-ME2 plays a role in basal defense against the hemibiotrophic fungal pathogen *Colletotrichum higginsianum*, and is involved in the generation of ROS (Voll et al., 2012), whereas *FQR1* is a primary auxin-response gene (Laskowski et al., 2002) that belongs to quinone reductases, classified as phase II detoxification enzymes, that protect organisms from oxidative stress; interestingly, *FQR1* acts as a susceptibility factor to *B. cinerea* (Heyno et al., 2013).

PTM-mediated regulation of oxidative-stress related proteins is in agreement with the notion that the redox state of the cell is dramatically altered in response to OGs.

### Other proteins

JAL34 and JAL27 (Table 2) are jacalin lectin-like proteins similar to myrosinase-binding protein 1 (Yamada et al., 2011) and to the functionally related PYK10. The latter is a β-glucosidase localized in the endoplasmic reticulum (ER) body, a large compartment specific to the Brassicales. PYK10 forms complexes with JALs and other proteins when tissue is damaged, i.e., upon herbivore or pathogen attack. Engagement in a complex may shield active PYK10 from inhibitors, proteases and other proteins that may reduce its defensive effect (Yamada et al., 2011). JAL34 and JAL27 show also OG-induced increased abundance at a later time point (Casasoli et al., 2008). JAL34 and JAL27 as well as PCaP1, ACT7, PATL2, and MDAR2 have been described in a previous proteomic work as proteins induced by brassinosteroids (Tang et al., 2008); whether these proteins are involved in the BR-mediated inhibition of PTI is still unknown.

## Conclusions

In conclusion, we have provided a large-scale study of early phosphoproteome changes in Arabidopsis following OG perception. In a recent phosphoproteomic study of Arabidopsis seedling total extracts following OG treatment, 51 phosphorylated peptides, representing 49 unique proteins, were identified (Kohorn et al., 2016). A comparison with our study shows only 9 shared phosphosites, corresponding to 9 proteins (Table 1), two of which also found in flg22-regulated phosphoproteome. Taking into consideration the other 16 shared flg22-regulated phosphosites, our work performed also on membrane proteins uncovers the immune-related regulation of 73 phosphosites. Protein interaction network analyses point to the main biological processes in which protein phosphorylation events are crucial in OG signaling, and suggest the interplay of several processes, e.g., intracellular trafficking and vesicle dynamics, cytoskeleton rearrangement, signal transduction and phospholipid signaling.

## Author contributions

BM, FS, and DP performed all the experiments and analyzed the data; BM and DP performed the LC-MS/MS analyses; BM and FS performed the 2D-DIGE analyses; BM and GD conceived the project and wrote and revised the article.

## Funding

This work was supported by the European Research Council (ERC Advanced Grant233083), the Italian Ministry of Agriculture Food and Forestry Policies (MIPAAF, Project ALISAL, DM 11008/7303/10) and University of Rome Sapienza (ATENEO, 2014).

### Conflict of interest statement

The authors declare that the research was conducted in the absence of any commercial or financial relationships that could be construed as a potential conflict of interest.
