# Supplementary material for: Comprehensive Analysis of the Membrane Phosphoproteome Regulated by Oligogalacturonides in Arabidopsis thaliana
Source: Front Plant Sci. 2016 Aug 2;7:1107. doi: 10.3389/fpls.2016.01107 (PMC4969306; doi:10.3389/fpls.2016.01107)
Supplement: Supplementary file 1 [file Table3.DOCX]

Supplementary Table S3. DIGE experimental design^a^.

Total extract

|  | Cy3 | Cy5 | Cy2 |
| --- | --- | --- | --- |
| Gel 1  Gel 2  Gel 3 | T3  C2  T2 | C1  T1  C3 | IS  IS  IS |
| Total microsomal fraction: |  |  |  |
| Gel 4  Gel 5  Gel 6 | T3  C2  T2 | C1  T1  C3 | IS  IS  IS |

^a^ T: OG-treated samples; C: H_2_O-treated samples (control). IS: Internal standard (a pool of C1+C2+C3+T1+T2+T3).
