# Supplementary material for: Comprehensive Analysis of the Membrane Phosphoproteome Regulated by Oligogalacturonides in Arabidopsis thaliana
Source: Front Plant Sci. 2016 Aug 2;7:1107. doi: 10.3389/fpls.2016.01107 (PMC4969306; doi:10.3389/fpls.2016.01107)
Supplement: Supplementary file 2 [file Table4.DOC]

Supplementary Table S4. Phosphosites differentially regulated by both OGs and flg22

| **Protein name^a^** | **TAIR ID^b^** | **Loc^c^** | **Uniprot ID^d^** | **Pos^e^** | **AA^f^** | **OG^g^** | **Flg22^h^** |
| --- | --- | --- | --- | --- | --- | --- | --- |
|  |  |  |  |  |  |  |  |
| Transcription regulator NOT2/NOT3/NOT5 family protein | At5g18230 | NU | F4JWJ6 | 431 | S | Up | Up |
| Probable serine/threonine-protein kinase | At1g18390 | PM | P0C5E2 | 643 | S | Down | Down |
| Patellin-4 (PATL4) | At1g30690 | PM | Q94C59 | 53 | S | Up | Up |
| Kinesin-like protein KAC1 | At5g10470 | CY | Q9LX99 | 698 | T | Up | Up |
| Enhancer of mRNA-decapping protein 4 | At3g13300 | CY | Q9LTT8 | 98 | S | Up | Up |
| NSP (Nuclear shuttle protein)-interacting GTPase | At4g13350 | CY | Q8W4K6 | 407 | S | Down | Down |
| Serine/arginine-rich splicing factor 30 | At3g55460 | NU | Q8L3X8 | 5 | S | Up | Up |
|  |  |  |  | 10 | S | Up | Down |
| Mitogen-activated protein kinase 6 (MPK6) | At2g43790 | CY | Q39026 | 223 | Y | Up | Up |
|  |  |  |  | 221 | T | Up | Up |
| ATPase 2, plasma membrane-type | AT4G30190 | PM | P19456 | 899 | S | Up | Up |
| ATPase 1, plasma membrane-type | AT2G18960 | PM | P20649 | 899 | S | Up | Up |
| ABC transporter G family member 36 (PEN3) | At1g59870 | PM | Q9XIE2 | 40 | S | Up | Up |
| Respiratory burst oxidase homolog protein D | At5g47910 | PM | Q9FIJ0 | 343 | S | Up | Up |
|  |  |  |  | 347 | S | Up | Up |
| Aluminium induced protein with YGL and LRDR motifs | At5g43830 | CY | Q9FG81 | 18 | S | Up | Up |
| Phospholipase like protein (PEARLI 4) family | At4g38550 | NU | Q9C5F6 | 41 | S | Down | Down |

**^a^** Full name of the identified protein..

**^b^** ID of the identified protein from the TAIR database (The Arabidopsis Information Resource database. www.arabidopsis.org).

**^c^** Subcellular localization, obtained from SUBA (the SUBcellular localization database for Arabidopsis proteins, http://suba3.plantenergy.uwa.edu.au/). PL: plastid, EX: extracellular, CY: cytosol, PM: plasma membrane, ER: endoplasmic reticulum, GO: Golgi apparatus, VO: vacuole, NU: nucleus, PX: peroxisome, MI: mitochondrion, CS: cytoskeleton.

**^d^** ID of the identified protein, from the UniProtKB database (<http://www.uniprot.org/>).

**^e^** Position of the phosphorylated amino acid on the whole sequence protein.

**^f^** The phosphorylated amino acid.

**^g^** Up and Down indicate increases and decreases in phosphorylation, respectively, upon OG treatment.

**^h^** Up and Down indicate increases and decreases in phosphorylation, respectively, upon flg22 treatment.
