# Supplementary material for: Comprehensive Analysis of the Membrane Phosphoproteome Regulated by Oligogalacturonides in Arabidopsis thaliana
Source: Front Plant Sci. 2016 Aug 2;7:1107. doi: 10.3389/fpls.2016.01107 (PMC4969306; doi:10.3389/fpls.2016.01107)
Supplement: Supplementary file 3 [file Table5.DOC]

Supplementary Table S5. OG-regulated phosphoproteins that have been identified as targets of MPK3/MPK6

| **TAIR ID^a^** | **Protein name^b^** |
| --- | --- |
| At4g38550 | Arabidopsis phospholipase-like protein (PEARLI 4) family |
| At4g02520 | ATGSTF2, ATPM24.1, ATPM24, GST2, GSTF2 - glutathione S-transferase PHI 2 |
| At5g11670 | ATNADP-ME2, NADP-ME2 - NADP-malic enzyme 2 |
| At3g19420 | ATPEN2, PEN2 - PTEN 2 |
| At5g50920 | CLPC, ATHSP93-V, HSP93-V, DCA1, CLPC1 - CLPC homologue 1 |
| At3g60240 | EIF4G, CUM2 - eukaryotic translation initiation factor 4G |
| At1g07110 | F2KP, ATF2KP, FKFBP - fructose-2,6-bisphosphatase |
| At5g02500 | HSC70-1, HSP70-1, AT-HSC70-1, HSC70 - heat shock cognate protein 70-1 |
| At5g52640 | HSP81-1, ATHS83, HSP81.1, HSP83, ATHSP90.1, AtHsp90-1, HSP90.1 - heat shock protein 90.1 |
| At3g13530 | MAPKKK7, MAP3KE1 - mitogen-activated protein kinase kinase kinase 7 |
| At4g13350 | NIG - NSP (nuclear shuttle protein)-interacting GTPase |
| At2g20960 | pEARLI4 - Arabidopsis phospholipase-like protein (PEARLI 4) family |
| At4g11850 | PLDGAMMA1, MEE54 - phospholipase D gamma 1 |
| At1g59870 | PEN3, PDR8, ATPDR8, ABCG36, ATABCG36 - ABC-2 and Plant PDR ABC-type transporter family protein |
| At4g39680 | SAP domain-containing protein |
| At3g11820 | SYP121, AT-SYR1, ATSYP121, SYR1, ATSYR1, PEN1 - syntaxin of plants 121 |
| At4g28080 | Tetratricopeptide repeat (TPR)-like superfamily protein |
| At4g02510 | TOC159, TOC86, PPI2, TOC160, ATTOC159 - translocon at the outer envelope membrane of chloroplasts 159 |
| At5g18230 | transcription regulator NOT2/NOT3/NOT5 family protein |
| At1g20970 | unknown protein |
| At3g13300 | VCS - Transducin/WD40 repeat-like superfamily protein |
| At1g78900 | VHA-A - vacuolar ATP synthase subunit A |

**^a^** ID of the identified protein from the TAIR database (The Arabidopsis Information Resource database. www.arabidopsis.org).

**^b^** Full name of the identified protein..
