# Supplementary figures and images for: Comprehensive Analysis of the Membrane Phosphoproteome Regulated by Oligogalacturonides in Arabidopsis thaliana
Source: Front Plant Sci. 2016 Aug 2;7:1107. doi: 10.3389/fpls.2016.01107 (PMC4969306; doi:10.3389/fpls.2016.01107)

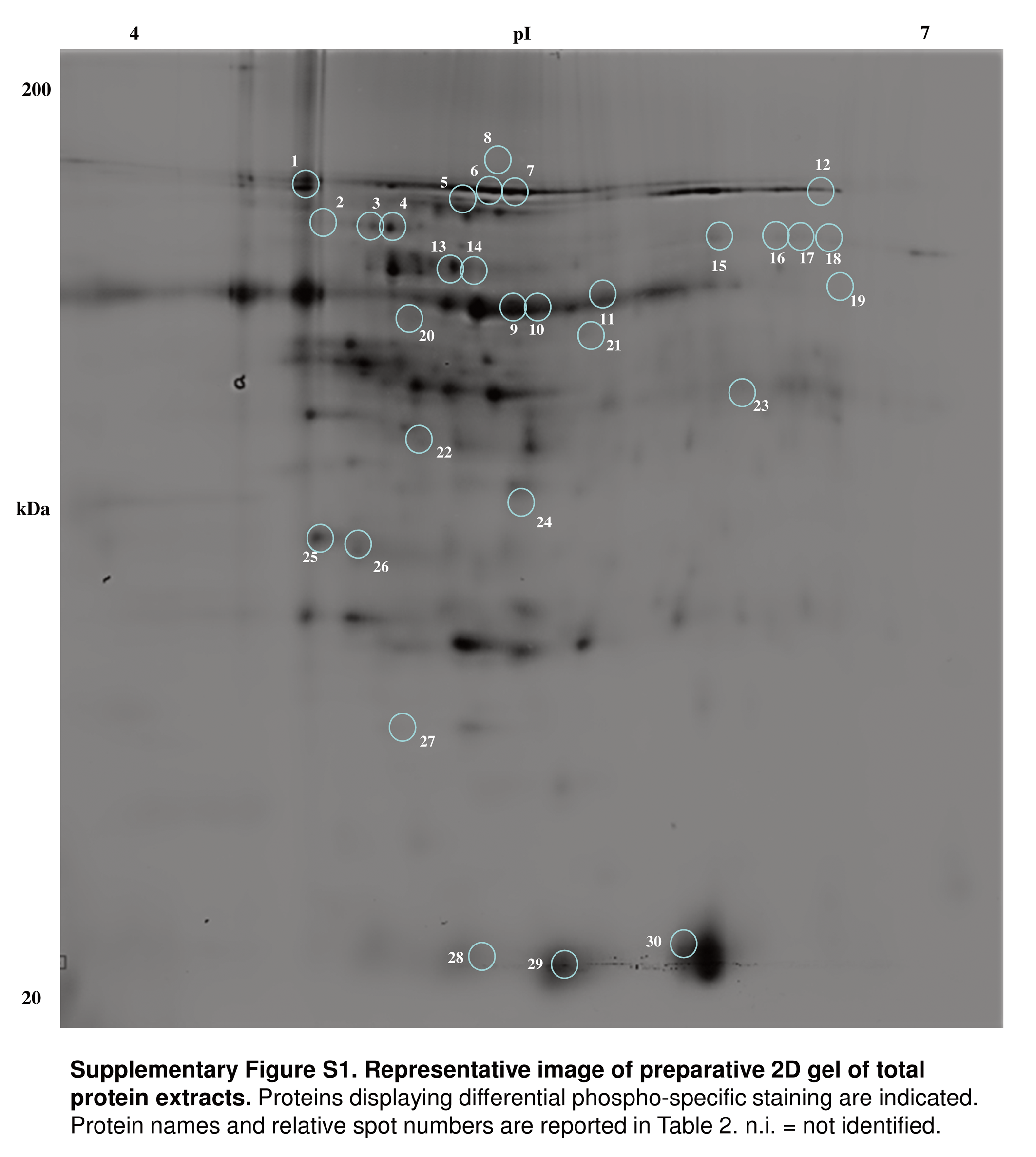

Supplement: Supplementary file 7 [file Image1.TIF]

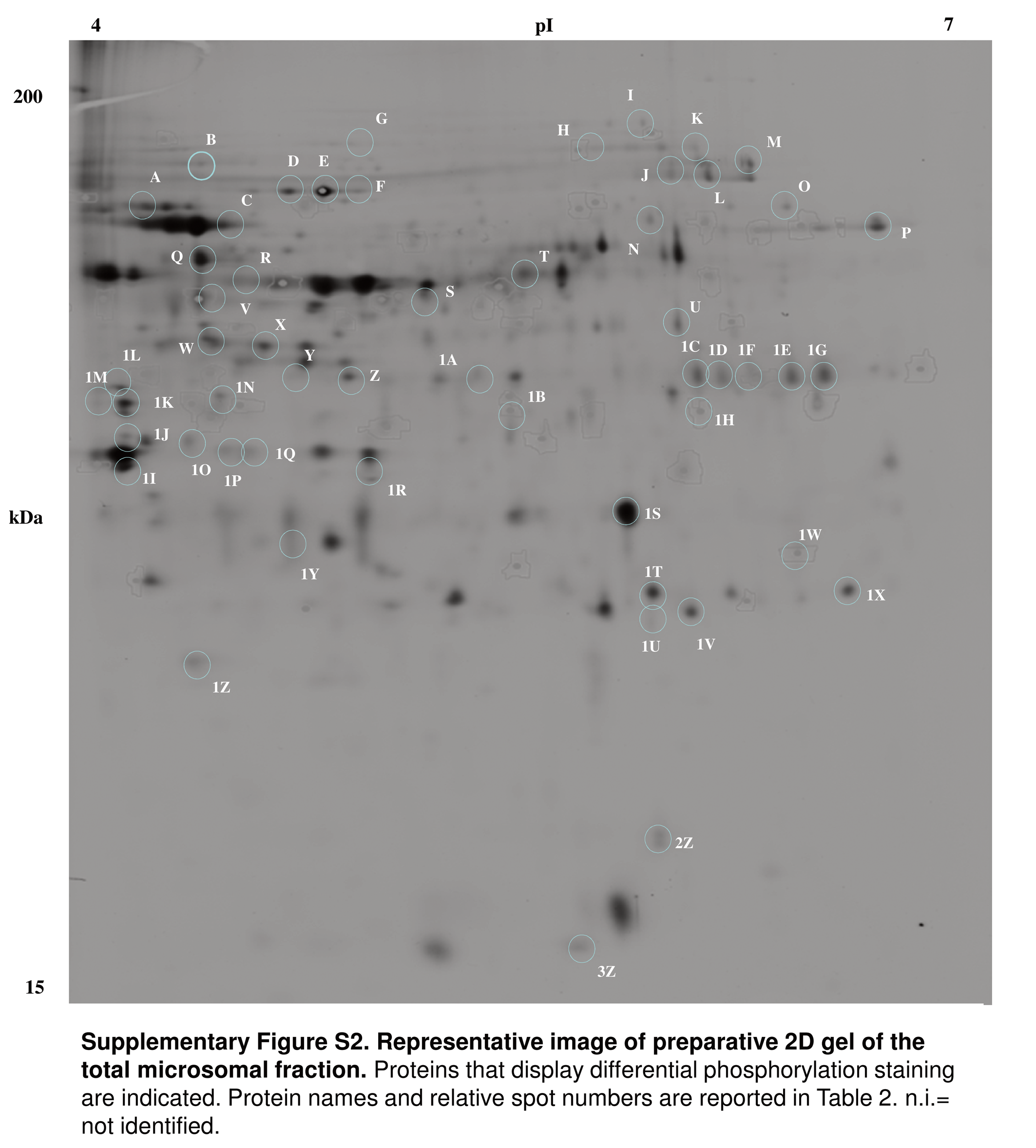

Supplement: Supplementary file 8 [file Image2.TIF]
